# Clinical Signs as an Indication of the Presence of Gastric Ulcers in Horses: True or False?

**DOI:** 10.3390/ani16182880

**Published:** 2026-09-13

**Authors:** Sara Busechian, Dania Cingottini, Micaela Sgorbini, Simona Orvieto, Irene Nocera, Francesco Zappulla, Fabrizio Rueca

**Affiliations:** 1 Department of Experimental Medicine, Faculty of Medicine and Surgery, University of Rome Tor Vergata, 00133 Rome, Italy; 2 Department of Veterinary Sciences, University of Pisa, 56124 Pisa, Italy; dania.cingottini@phd.unipi.it (D.C.); micaela.sgorbini@unipi.it (M.S.); irene.nocera@unipi.it (I.N.); 3 Private Practitioner, 06100 Perugia, Italy; simona.orvieto@gmail.com (S.O.); frueca@gmail.com (F.R.); 4 Ministry of Health, Directorate-General for Animal Health, 00144 Rome, Italy; f.zappulla@sanita.it

**Keywords:** equine squamous gastric disease, equine glandular gastric disease, clinical signs, signalment, pain perception

## Abstract

Equine Gastric Ulcer Syndrome (EGUS) and its related diseases, Equine Squamous Gastric Disease (ESGD) and Equine Glandular Gastric Disease (EGGD), can be found in horses worldwide, with increased prevalences in racehorses and animals subjected to heavy training loads. Clinical signs of the disease are vague and nonspecific, and most horses affected by the disease are asymptomatic. In this retrospective study, our aim was to determine the percentages of pleasure and breeding horses showing signs compatible with gastric ulcers, and to correlate these signs with the presence of ESGD, EGGD, and both diseases at the same time. The effects of signalment and activity on disease development and symptoms were also investigated. In total, 1125 adult horses were enrolled in the study. Within this group, only 12% showed clinical signs related to the gastrointestinal tract (GIT, 6%), performance and behavior (PER, 1%), or weight loss (WL, 5%). ESGD was diagnosed in 63% (grade 2/4 22%, 3/4 11%, and 4/4 30%) of animals, while EGGD was diagnosed in 32%. Warmblood horses and geldings were more likely to show clinical signs compared to the other breeds and sexes. WL was associated with the presence and severity of ESGD and the presence of EGGD; GIT was associated only with the presence and severity of ESGD; and PER was associated with the severity of ESGD and the presence of EGGD. Activity was the main risk factor for the development of all diseases, modulating the effect of signalment. EGGD was the only disease with a limited effect on the manifestation of clinical signs when compared with the effect of signalment only. Despite the relatively high prevalence of the disease in horses considered at low risk, clinical signs were present in only a small percentage of the study group, with their expression possibly related to the presence and severity of gastric ulcers. Although the limited effect of EGGD on the manifestation of clinical signs needs to be confirmed, it highlights the limited knowledge we currently have on the clinical significance of lesions in the glandular mucosa, especially when only hyperemia is present.

## Simple Summary

Gastric ulcers, a disease affecting horses and various other categories of animals worldwide, seem to be related, for the most part, to management conditions and exercise. Despite the high prevalence of gastric ulcers in horses, described in the literature, most horses show no overt clinical signs of the disease, and symptoms are often vague and nonspecific when they are present. In this study, our objectives were to evaluate the presence of clinical signs in 1125 adult horses diagnosed with gastric ulcers, to identify whether specific symptoms are related to the position of lesions (squamous or glandular gastric mucosa) or their severity, and to determine whether the sex, age, or breed of the horse influences the clinical manifestation of the disease. While the number of horses showing clinical signs in this population was very small, at only 12%, the category of symptoms could indicate the presence and severity of the disease. Warmblood geldings were more likely to demonstrate signs compared to other breeds and sexes. This study confirms the high number of horses presenting gastric ulcers without any outside manifestation of the disease, while also showing that the presence of symptoms can be indicative of lesions. A thorough clinical examination, including gastroscopy, should be performed to help guide decisions on the best treatment and management plan for these animals.

## 1. Introduction

Equine Gastric Ulcer Syndrome (EGUS) is defined as the presence of erosions or ulcers on the gastric mucosa. In 2015, the European College of Equine Internal Medicine released a Consensus Statement that divided EGUS into two different diseases—Equine Squamous Gastric Disease (ESGD) and Equine Glandular Gastric Disease (EGGD)—one for each of the two mucosae in which the stomach lining is divided: squamous and glandular, respectively [1,2,3,4,5,6]. This division was made due to the different epidemiology, risk factors, pathophysiology, and response to treatment of the two diseases. ESGD seems to be caused mostly by the management of horses (amount of carbohydrates in the diet, number of meals per day, transportation, etc.) [1,3,5,6,7,8], while EGGD is related to intrinsic factors of the horse, such as temperament and response to stress [1,2,3,6,7,8]. The clinical signs are usually nonspecific with either disease and can include ill-thrift, weight loss, and recurrent colic, while many horses show no overt symptoms. Within the last few years, associations between behavioral problems and EGUS have been proposed [9,10], and the role of gastric ulcers in poor performance has been thoroughly investigated [11,12,13,14,15]. These studies have contributed to an increasing amount of information on the possible negative effects of gastric ulcers on performance, not only from a fitness point of view but also from a behavioral one [16]. The number of asymptomatic horses, however, raises questions about the clinical significance of the disease, especially at lower severities [1,2]. Two recent studies have found that clinical signs are not good predictors of the disease, while the most common complaint is related to behavioral problems, especially during saddling [16,17,18]. Although girthiness has long been associated with gastric ulcers, due to its aspecific nature, a definitive diagnosis of the disease requires gastroscopy [19]. On the other hand, the effect of signalment on pain expression in horses has not yet been completely elucidated, and it is not clear how the presence of gastric ulcers could modulate how different sexes, breeds, and age groups show discomfort.

In this retrospective study, our objectives were to examine the presence of owner-reported clinical signs in a population of horses subjected to gastroscopic examination between 2013 and 2026, and to determine if the presence and type of symptoms could be related to the presence and severity of ESGD and EGGD. Furthermore, in this population, we sought to determine if signalment and the presence of gastric lesions could influence the manifestation of discomfort.

## 2. Materials and Methods

### 2.1. Animals

Horses of at least 1 year of age, submitted for gastroscopic examination at the request of referring veterinarians, owners, and trainers, were included in this retrospective study. Animals were excluded if they were subjected to heavy training loads (i.e., racehorses, sport horses during the peak of the season), if they were diagnosed with or showed signs of any disease other than EGUS, or if they were being treated with any medication at the time of the gastroscopy. Horses were also excluded if they had been treated with omeprazole or other gastroprotectants in the three months before the examination. Endoscopy was performed either as part of a complete diagnostic protocol in horses presenting with clinical signs or as a single examination to determine the prevalence of EGUS in pleasure and breeding horses. Informed consent was provided by all owners or caretakers. Before the endoscopy, owners and caretakers were asked if their animals presented any clinical sign that could be related to gastric ulcers (i.e., recurrent colic, behavioral problems, poor body condition score). The responses were recorded in an Excel sheet, together with information about the signalment of the horse and the type of activity or sport they were performing at the time of the examination.

### 2.2. Gastroscopy

Gastroscopy was performed according to the literature, using a 3 m long endoscope (60130PKS, Karl Storz, Tuttlingen, Germany) and a portable processor (Tele pack Vet X LED, Karl Storz, Tuttlingen, Germany) [7,20]. A clinical examination was conducted, during which the horses were sedated with xylazine (0.5–1 mg/kg) and restrained with a twitch. The scope was inserted through the left nostril and passed through the pharynx and the esophagus to reach the stomach. The organ was inflated with air and examined, cleaning the mucosa with sterile water when needed. The video and still images of the examination were recorded on a USB stick for offline scoring of EGUS and archiving. The presence of lesions on the squamous lining was graded according to the ECEIM Consensus Statement guidelines, using a 5-point scale. Meanwhile, due to the lack of a validated scoring system for EGGD, alterations were only described, and animals were considered positive for EGGD if they showed changes in the appearance of their glandular mucosa [1,2,7]. Horses diagnosed with at least grade 2 ESGD and EGGD were considered positive for EGUS.

### 2.3. Statistical Analysis

All the raw data (signalment, clinical signs, scoring and presence of gastric ulcers, activity) were collected in an Excel spreadsheet for the analysis and evaluation of the information’s coherence. To reduce the number of variables, breeds were grouped into five categories: Fullbloods (Arabians, Thoroughbreds, Standardbreds, Quarter Horses, and related breeds), Warmbloods (all saddle breeds), Baroque (Lipizzaners, Spanish purebreds, Murgese, Friesians), Coldbloods (draft horses, Haflingers), and Ponies [21]. Horses were also grouped by age: colts and fillies (4 years old or less), young (5–10 years of age), adult (11–20 years of age), and old (21 years and above) [22]. The main clinical complaint was the one that was considered, and clinical signs were retrospectively categorized as “weight loss”, “gastrointestinal” (i.e., recurrent colic), or “performance/behavioral-related” (e.g., poor performance, girthiness, nervousness while being ridden, etc.). Weight loss and poor body condition scores were evaluated and confirmed by the examining veterinarians at the time of the clinical examination before the gastroscopy. The prevalences of ESGD, EGGD, and EGUS were calculated, together with the distribution of animals in the categories for breed, sex, and age, and in groups for clinical signs and activity. The numbers of animals affected by different degrees of severity of ESGD were also determined. Linear and logistic models were used to correlate the presence and severity (e.g., the different grades of ESGD) of gastric ulcers with signalment, the presence and category of clinical signs, and the activity performed by the horse. For the multiple models, the Likelihood Ratio Test and Chi-Square were used to compare baseline models that only included signalment with those that also included activity and clinical signs to determine if these additions improved the fit. Additional models, both univariate and multivariate, were used to determine if the presence of clinical signs was influenced by the signalment alone or the given ESGD, EGGD, and EGUS status. Odds ratios and 95% confidence intervals were calculated, with statistical significance set at *p* < 0.05. Analysis was performed using RStudio (version 2025.09.1) [23].

## 3. Results

**Figure 1:**
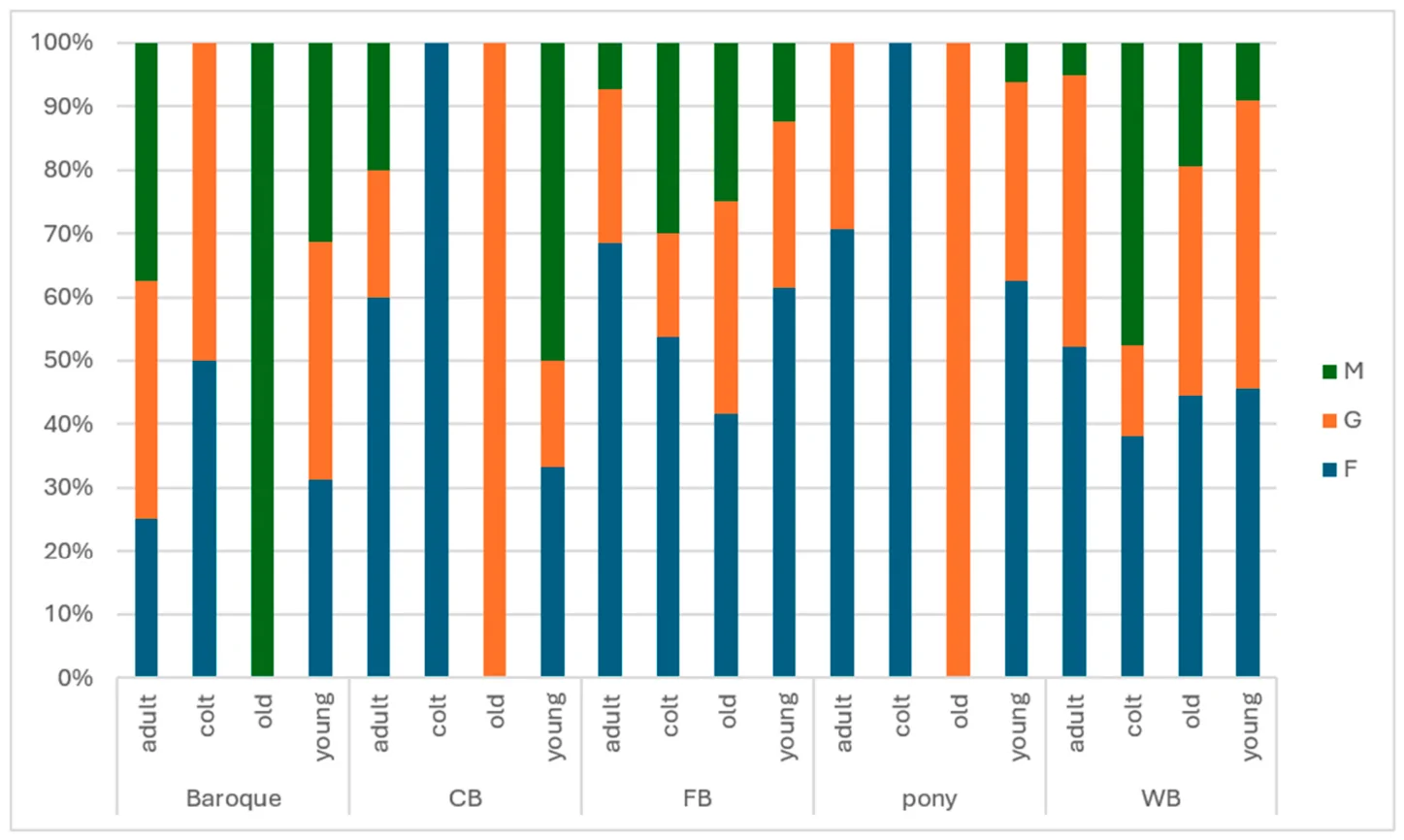
Distribution of the horses enrolled in the study based on age, breed group, and sex. Adult: 11–20 years of age; colt (and fillies): 4 year old or less; old: 21 years and above; young: 5–10 years of age. M: male; G: gelding; F: female. Baroque: Lipizzaners, Spanish purebreds, Murgese, Friesians; CB, Coldblood: draft horses, Haflingers; FB, Fullblood: Arabians, Thoroughbred, Standardbred, Quarter horses, Paint and Appaloosa; Pony: Welsh Pony, Shetland; WB, Warmblood: KWPN, German saddlebred, Zangersheide, Italian saddlebred, French Saddlebred.

A total of 1125 horses were included in the study, aged between 1 and 30 years of age (median 10.5 yo, IQR 6–14 yo). Based on the age groups identified in the statistical analysis, 105/1125 horses were 4 years of age or younger (9%), 497/1125 were young (44%), 472/1125 were adult (42%), and 51/1125 were old (5%). All sexes were represented, with most horses being female (619/1125, 55%), followed by geldings (370/1125, 33%) and males (136/1125, 12%). In terms of breed, an almost equal number of Fullbloods (521/1125, 46%) and Warmbloods (504/1125, 45%) were present, with less Ponies (52/1125, 5%), Baroque (35/1125, 3%), and Coldbloods (13/1125, 1%). Most of the horses were used for English riding, mainly showjumping (413/1125, 37%); 256/1125 (23%) were breeding animals, both males and females; and 168/1125 (15%) were kept as pets, without being employed in any kind of activity. Others were used for Western riding (112/1125, 10%), trekking (77/1125, 7%), endurance (32/1125, 3%), Arabian halter competitions (21/1125, 2%), and as draught horses (7/1125, 1%). For the remaining 39/1125 (3%), the activity was categorized as “Other”, which included all sports and uses with only one or two horses, such as equine-assisted therapies, working equitation, etc. The distribution of the animals according to signalment is shown in Figure 1.

**Figure 2:**
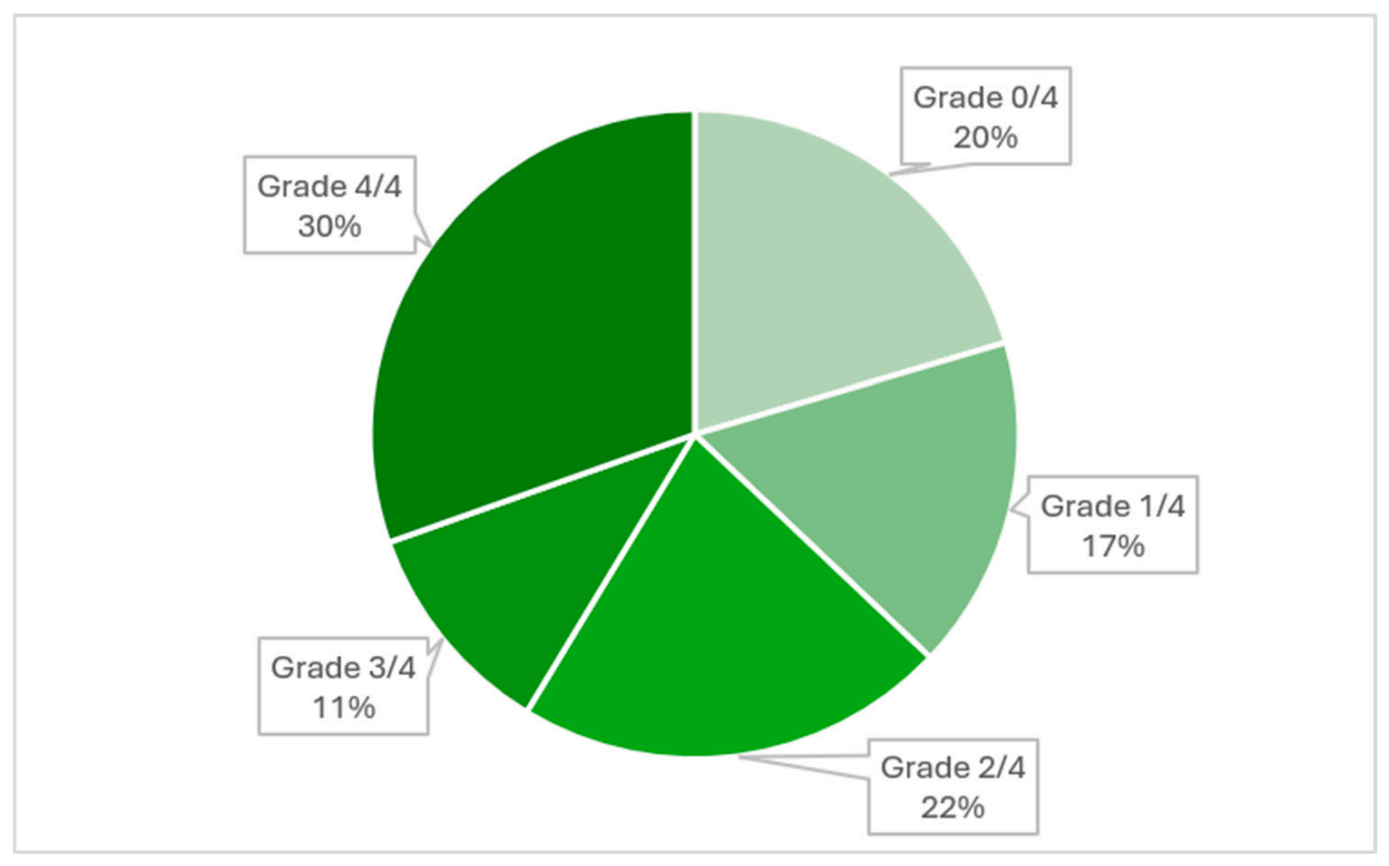
Distribution of the grades of ESGD in the population of 1125 horses included in the study.

A total of 708/1125 horses were positive for ESGD (grade 2 and above) (63%). EGGD was diagnosed in 359/1125 horses (32%): lesions were limited to areas of hyperemia of variable sizes on the larger curvature, sometimes round and sometimes linear, and no erosions or ulcerations were recorded. The number of EGUS-positive horses, those that were diagnosed with both ESGD and EGGD, was 315/1125 (28%). The distribution of the animals according to grades of ESGD is shown in Figure 2. Table 1 shows the distribution of EGGD in animals diagnosed with different grades of ESGD.

According to the owners, clinical signs were present in 132/1125 horses (12%): 64/1125 (6%) had signs related to the gastrointestinal tract and 15/1125 (1%) had signs related to performance and behavior. Poor body condition and weight loss were found in 53/1125 (5%) horses: these changes were described by the owners and confirmed by veterinarians during the clinical examination before the gastroscopy. Table 2 shows the allocation of horses with and without clinical signs for each grade of ESGD, while Table 3 lists presence of clinical signs in animals diagnosed with EGGD.

Based on the univariate analysis, both presence and severity of ESGD was correlated with age group, sex, and activity. Lesions of the squamous mucosa were also associated with the presence of clinical signs in general, and also with the three types of symptoms (weight loss, gastrointestinal tract, and behavior). EGGD was determined by breed, age group, sex, and activity, and was related to the presence and type of clinical signs. The presence of lesions in both mucosae at the same time was correlated with breed, age group, sex, and activity, but also with the presence and type of clinical signs. The multivariate analysis showed that presence of ESGD, EGGD, and both diseases at the same time and the severity of ESGD were determined by age group and sex. Adding activity to the basal model improved the fit of all (*p* < 0.001). The statistically significant results of the univariate and multivariate models can be found in Table 4, Table 5, Table 6, Table 7 and Table 8, while the full models are reported in Supplementary Materials Tables S1 and S2.

Clinical signs were associated with the presence of all diseases and with the severity of ESGD. Weight loss was correlated with all diseases, while symptoms connected to altered behavior and poor performance were associated with EGGD, the severity of ESGD, and the presence of ESGD and EGGD at the same time. Gastrointestinal signs were associated with the presence and severity of ESGD and with EGGD. From a diagnostic point of view, combining activity, signalment, and clinical signs improved the ability of symptoms to diagnose ESGD, though a good fit was given by adding just activity to the basal model of signs. For EGGD and EGGD, the best fit was determined by the full model, which included signalment, activity, and clinical signs. These full models can be found in Table 9, Table 10, Table 11 and Table 12, while the complete results of this statistical analysis can be found in Supplementary Materials Table S3.

The presence and categories of symptoms were correlated with the breed, sex, and activity of the horses. The multivariate analysis confirmed these data but showed that horses with ESGD were more likely to show clinical signs. On the other hand, EGGD and the presence of both diseases at the same time showed less effect on the development of symptoms, while maintaining a statistically significant relationship. Table 13, Table 14, Table 15, Table 16 and Table 17 show the statistically significant analysis, while the full results can be found in Supplementary Materials Table S4.

## 4. Discussion

Gastric ulcers in both squamous and glandular mucosae have been diagnosed in horses worldwide, with different prevalences depending on the population examined [1,2,3,4,5,6,7,8,22,24,25,26]. Despite this widespread distribution, however, the clinical significance of the disease has not yet been completely elucidated due to the high number of affected horses not showing clinical signs [3,6].

In this study, the distribution of animals according to their signalment and activity reflects the current situation in Italy, where most horses are either Fullblood or Saddlebred and employed for English riding, especially showjumping.

In this population of 1125 horses not subjected to high-intensity training, the prevalence of ESGD (63%) was higher than in other similar populations, while EGGD (32%) was present at an almost comparable percentage [4,5,6,7]. The presence of ESGD, however, was similar to that described by another paper examining pleasure horses: almost 60% of animals included in the study that presented clinical signs had gastric ulcerations, though the study did not indicate which mucosa was affected [27]. The cause of the relatively high prevalence of ESGD in this study is not clear. Animals were included if they presented clinical signs or risk factors for gastric ulcers, but were also included at the request of the vets or owners of horses in the same premises of affected subjects. The high prevalence could be related to the type of activity performed by the majority of the animals: in a similar population of horses, English riding was considered a risk factor for the development of ESGD in a multivariate analysis that included the time spent training per week [7]. For showjumpers in Canada, the prevalence of ESGD grades 2 and above was 25%, significantly lower than in the horses studied here, while EGGD was slightly higher, at 51% [28].

The univariate and multivariate analyses of risk factors for all diseases confirm the findings within the current literature, with younger animals, especially males, being more at risk than older ones for both ESGD and EGGD and for the concurrent presence of lesions in both mucosae [1,2,3,6,7]. Activity seems to have the greatest effect on the development of the diseases, even in this population of horses not subjected to high-intensity training. In particular, hand halter competitions, which in this population were performed mostly by Arabians, increased the risk of ESGD, while at the same time being protective for the development of EGGD. Although information on gastric ulcers in this category of animals is scarce in the literature, the type of management they are subjected to could be responsible for the increased risk of lesions in the squamous mucosa. These horses are usually fed high quantities of grains, while at the same time being exercised and usually maintained with minimal access to paddocks [29]. It has been demonstrated that management, especially management similar to that described for this category of animals, has a greater effect on ESGD compared to EGGD, where the major determinants are factors related to the horse, especially when considering pleasure animals and those not subjected to high-intensity training [7].

Clinical signs of gastric ulcers in horses are vague and nonspecific, ranging from ill-thrift to poor performance and recurrent colic, with many animals showing no overt symptoms [1,2,3]. This is confirmed by the population in this study, in which only 12% of horses were symptomatic. Poor performance was scarcely present (only 1%), though this could have resulted from the fact that none of the animals were involved in heavy training or high-level performances, making it more difficult for the owners to detect subtle changes in results. Despite the low number of horses presenting clinical signs, symptoms can be considered good predictors of the presence of lesions—particularly weight loss and poor body condition score at clinical examination. Poor performance, on the other hand, was not associated with ESGD but only with EGGD, confirming the significant impact of lesions in the glandular mucosa on the welfare of horses. It needs to be stressed, however, that, in this population, the level of activity could have led to an under-representation of this complaint, especially when considering that a high number of animals were not ridden or trained but were either kept as pets or used for breeding. Some owners could also not be aware of rideability issues at the time of gastric ulcer diagnosis and could become aware of improvements in behavior after healing. A recent study found that, after the treatment of EGUS, Ridden Horse Pain Ethogram scores decreased, indicating a general improvement in the wellbeing of ridden animals after ulcer resolution [30]. Another paper found that, after the healing of ESGD and EGGD, trained showjumping horses showed an increased maximal stride length and speed, with no change in concurrent heart rate, confirming that a decrease in abdominal discomfort, secondary to ulcer healing, improved horse wellbeing while ridden [15]. Gastrointestinal signs, such as recurrent colic or diarrhea, were associated with both ESGD and EGGD. These results are in line with the current literature: in recent years, several papers have been published on the presence of owner-reported clinical signs and their association with gastric ulcers [17,18,31]. In these papers, behavioral problems and gastrointestinal signs were common in horses with ESGD and EGGD. One study on 52 animals found that physical symptoms, including recurrent colic, diarrhea, and ill-thrift, were more common in horses with squamous lesions, while glandular ulcers were associated with behavioral changes, such as poor performance, irritability, and poor rideability [31]. In a study of 850 horses suspected of having EGUS, a group of healthy controls showed behavioral symptoms in a percentage similar to that of the diseased ones, highlighting the importance of confirming EGUS through gastroscopy when horses present with rideability issues [18]. Furthermore, the authors of the present study have found that owners and referring vets more often request gastroscopic examination for behavioral problems, especially when saddling or during exercise, or treat the animals with gastroprotectant drugs for these issues without gastroscopic examination, and this trend is increasing in recent years.

The manifestation of clinical signs in this population seems to be modulated not only by the presence of gastric ulcers, but also by the signalment of the animals: Warmblood geldings seem to present symptoms more commonly than Fullblood females. To the best of the authors’ knowledge, there is little information in the literature on differences in the perception of pain and discomfort across the different sexes in horses, while, in humans, this variance is well documented, with women being more prone to perceive and manifest pain compared to men due to hormonal influences. Women are also more frequently subjected to chronic pain compared to men [32]. These results, however, are in contrast with the data from this population of horses, and further studies are needed to confirm these findings, which can only be speculative at this stage.

Regarding the effect of disease presence on the presence of clinical signs, it is interesting to note that, in the multivariate analysis, grades 2 and above significantly increased the odds of showing symptoms, with a higher probability associated with higher grades (grade 2 OR 2.446, 3 OR 5.118 and 4 OR 6.922), confirming the clinical significance of increased severity [1,3,5]. It is also interesting to note that EGGD does not modulate the manifestation of clinical signs of gastric lesions as much as the severity of ESGD, as indicated by the results of the Chi-Square test (11.15 vs. 58.59, respectively). In this population, however, EGGD was present only as areas of hyperemia of various extension: the clinical significance of glandular lesions is at the moment not completely clear. Based on these results, we could postulate that hyperemia, especially when small in size or only present in certain areas of the stomach, could have limited effect on the development of clinical signs, and could therefore potentially not need treatment, as currently advised for animals with ESGD grade 1 [1,3,5]. The lack of severe lesions in the glandular mucosa could potentially limit the results of this present study only to mild forms of EGGD, showing the importance of further studies including more severe cases of glandular lesions. It is important to bear in mind, however, that some horses with ESGD grade 1–4 (hyperkeratosis) could still present clinical signs and respond favorably to treatment [3].

This study has several limitations. Clinical signs were reported by the owners and not confirmed by the authors, except for poor body condition score. This, along with the fact that horses were included at request of trainers when stabled in the same premises as those presenting with gastric ulcers, could potentially present a selection bias. Furthermore, only the main sign was recorded. The number of horses showing more than one symptom was negligible, usually represented by either poor performance or behavioral issues, which in this study were grouped together. While this approach has been used in other studies, poor performance and girthiness could potentially be caused by lesions in different areas of the stomach due to the different physiology and pathophysiology of the two gastric mucosae: the glandular highly specialized to resist acid exposure, and the squamous less protected but more exposed to gastric fluid, especially during exercise [33]. Another limitation is the lack of information on the resolution of clinical signs after the treatment and healing of gastric lesions. This was due to the retrospective nature of the study: owners often refused a follow-up examination, considering it invasive and not necessary, especially in animals that, according to their perception, were doing well. Some owners, on the other hand, despite the presence of gastric ulcers, declined treatment in horses without clinical signs due to the cost of the drug, preferring to use supplements and to monitor their animals for clinical signs. Finally, the authors chose to exclude horses in heavy training from this study: this team has focused in the last decade on pleasure and breeding horses, and the number of racehorses and elite riding animals scoped was limited due to constraints related to doping, owners’ concerns about the procedure, and schedule conflicts (travel and shows or races). Limiting the study to low-level exercise horses could have underrepresented the number of animals showing behavioral or performance problems. However, as previously stated, owners’ perception of riding issues has changed over the last decade, with an increase in horses presented for gastroscopy due to girthiness or nervousness during saddling or exercise: these issues are now investigated for physical diseases, not as manifestations of the “horse being difficult”.

## 5. Conclusions

For the 1125 horses included in this study, ESGD and EGGD were present in a high percentage of the population despite a low number of animals with clinical signs. When present, symptoms were associated with the development of lesions in both mucosae of the stomach: weight loss was associated with ESGD and EGGD and with lesions in both linings; gastrointestinal signs were associated with ESGD and EGGD, but not with both diseases; and poor performance and behavioral issues were associated with EGGD alone or in combination with ESGD. The manifestation of clinical signs was influenced by the signalment of the horses and modulated mostly by the severity of ESGD, while EGGD had less of an influence on the development of symptoms.

Further studies are needed to confirm these results and to determine the effects of sex and breed on pain perception in horses.

## Figures and Tables

**Table 1:** Distribution of EGGD-positive and -negative horses according to grades of ESGD in the population of 1125 animals.

	Number of Horses
**ESGD grade 0**	**230**
EGGD-negative	217
EGGD-positive	13
**ESGD grade 1**	**187**
EGGD-negative	156
EGGD-positive	31
**ESGD grade 2**	**243**
EGGD-negative	167
EGGD-positive	76
**ESGD grade 3**	**123**
EGGD-negative	74
EGGD-positive	49
**ESGD grade 4**	**342**
EGGD-negative	152
EGGD-positive	190
**Total**	**1125**

**Table 2:** Distribution of clinical signs according to the different grades of ESGD in this population of 1125 horses.

	Number of Horses
**ESGD grade 0**	**230**
No clinical signs	221
Clinical signs	9
**ESGD grade 1**	**187**
No clinical signs	177
Clinical signs	10
**ESGD grade 2**	**243**
No clinical signs	221
Clinical signs	22
**ESGD grade 3**	**123**
No clinical signs	103
Clinical signs	20
**ESGD grade 4**	**342**
No clinical signs	271
Clinical signs	71
**Total**	**1125**

**Table 3:** Distribution of clinical signs in horses with and without EGGD in this population of 1125 animals.

	Number of Horses
**EGGD-negative**	**766**
No clinical signs	692
Clinical signs	74
**EGGD-positive**	**359**
No clinical signs	301
Clinical signs	58
**Total**	**1125**

**Table 4:** Results of the univariate analysis reporting only the statistically significant parameters. The full results can be found in Supplementary Materials Table S1. NA: not available.

Variable	Odds Ratio	2.5%	97.5%	*p*-Value
**PRESENCE OF ESGD**				
**Age groups**				
**Young**	1	NA	NA	Reference
**Colt/filly**	1.330	0.830	2.131	0.2360
**Adult**	0.658	0.506	0.854	0.0017
**Old**	0.430	0.240	0.768	0.0044
**SEX**				
**Female**	1	NA	NA	Reference
**Gelding**	1.003	0.770	1.307	0.9823
**Male**	1.671	1.108	2.521	0.0143
**Activity**				
**No activity**	1	NA	NA	Reference
**Driving**	0.261	0.050	1.369	0.1121
**Endurance**	0.738	0.353	1.545	0.4209
**English riding**	1.155	0.837	1.592	0.3805
**Show**	13.032	1.722	98.615	0.0129
**Trekking**	0.824	0.492	1.379	0.4615
**Western riding**	1.781	1.094	2.900	0.0203
**Other**	4.431	1.677	11.708	0.0027
**Presence of clinical signs**				
**No**	1	NA	NA	Reference
**Yes**	3.978	2.407	6.574	<0.001
**CATEGORIES OF CLINICAL SIGNS**				
**No clinical signs**	1	NA	NA	Reference
**Gastrointestinal signs**	3.223	1.663	6.246	0.001
**Poor performance**	3,851,444.569	0.000	Inf	0.9678
**Weight loss**	3.763	1.755	8.067	<0.001
**SEVERITY OF ESGD**				
**Age groups**				
**Young**	1	NA	NA	Reference
**Colt/filly**	1.438	0.975	2.120	0.0670
**Adult**	0.726	0.581	0.909	0.0051
**Old**	0.467	0.275	0.792	0.0047
**Sex**				
**Female**	1	NA	NA	Reference
**Gelding**	1.023	0.814	1.284	0.8478
**Male**	1.925	1.367	2.710	<0.001
**Activity**				
**No activity**	1	NA	NA	Reference
**Driving**	0.147	0.027	0.786	0.025
**Endurance**	0.728	0.369	1.436	0.3596
**English riding**	1.200	0.910	1.583	0.1962
**Show**	5.574	2.254	13.784	<0.001
**Trekking**	0.951	0.603	1.501	0.8297
**Western riding**	1.961	1.317	2.920	<0.001
**Other**	2.577	1.417	4.684	0.0019
**Presence of clinical signs**				
**No**	1	NA	NA	Reference
**Yes**	3.384	2.403	4.765	<0.001
**Categories of clinical signs**				
**No clinical signs**	1	NA	NA	Reference
**Gastrointestinal signs**	2.551	1.617	4.020	<0.001
**Poor performance**	12.030	3.437	42.104	<0.001
**Weight loss**	3.657	2.148	6.224	<0.001
**PRESENCE OF EGGD**				
**Breed**				
**Fullblood**	1	NA	NA	Reference
**Warmblood**	0.760	0.584	0.989	0.0409
**Baroque**	0.854	0.409	1.782	0.6736
**Coldblood**	0.339	0.074	1.544	0.1619
**Pony**	0.986	0.542	1.795	0.9635
**Age groups**				
**Young**	1	NA	NA	Reference
**Colt/filly**	1.943	1.270	2.972	0.0022
**Adult**	0.702	0.533	0.924	0.0116
**Old**	0.355	0.163	0.771	0.0089
**Sex**				
**Female**	1	NA	NA	Reference
**Gelding**	1.112	0.842	1.468	0.4531
**Male**	1.593	1.086	2.337	0.0173
**Activity**				
**No activity**	1	NA	NA	Reference
**Driving**	0.431	0.054	3.897	0.4769
**Endurance**	1.257	0.566	2.789	0.5744
**English riding**	1.403	0.994	1.979	0.0542
**Show**	0.651	0.211	2.002	0.4534
**Trekking**	1.178	0.672	2.064	0.5682
**Western riding**	1.999	1.254	3.188	0.0036
**Other**	4.424	2.192	8.928	<0.001
**Presence of clinical signs**				
**No**	1	NA	NA	Reference
**Yes**	1.802	1.245	2.607	0.0018
**Categories of clinical signs**				
**No clinical signs**	1	NA	NA	Reference
**Gastrointestinal signs**	1.474	0.876	2.479	0.1439
**Poor performance**	3.449	1.217	9.774	0.0199
**Weight loss**	1.903	1.089	3.323	0.0237
**PRESENCE OF ESGD AND EGGD AT THE SAME TIME**				
**Age groups**				
**Young**	1	NA	NA	Reference
**Colt/filly**	2.044	1.333	3.135	0.0011
**Adult**	0.643	0.482	0.859	0.0027
**Old**	0.358	0.158	0.812	0.014
**Sex**				
**Female**	1	NA	NA	Reference
**Gelding**	1.131	0.847	1.511	0.4044
**Male**	1.791	1.213	2.645	0.0034
**Activity**				
**No activity**	1	NA	NA	Reference
**Driving**	0.595	0.070	5.047	0.6343
**Endurance**	1.398	0.612	3.191	0.4269
**English riding**	1.497	1.040	2.155	0.0297
**Show**	0.840	0.272	2.598	0.7626
**Trekking**	1.339	0.748	2.398	0.3256
**Western riding**	2.143	1.321	3.477	0.002
**Other**	3.759	1.878	7.528	<0.001
**Presence of clinical signs**				
**No**	1	NA	NA	Reference
**Yes**	1.942	1.335	2.824	<0.001
**Categories of clinical signs**				
**No clinical signs**	1	NA	NA	Reference
**Gastrointestinal signs**	1.469	0.861	2.508	0.1587
**Poor performance**	4.207	1.483	11.933	0.0069
**Weight loss**	2.150	1.227	3.769	0.0075

**Table 5:** Multivariate analysis to determine the risk factors for the presence of ESGD. Comparison between the two models: “base”, with only signalment, and “full”, with signalment and activity. Ref: reference; NA: not available. Likelihood Ratio Test: activity on ESGD; Chi-Square statistic (weight): 36.16; *p*-value: <0.001.

	Base Model, Only Signalment				Full Model, with Activity			
	Odds Ratio	2.5%	97.5%	*p*-Value	Odds Ratio	2.5%	97.5%	*p*-Value
Fullblood	1	NA	NA	Ref	1	NA	NA	Ref
Warmblood	0.914	0.699	1.194	0.5076	0.954	0.678	1.343	0.7893
Baroque	0.587	0.289	1.189	0.1392	0.598	0.281	1.274	0.1829
Coldblood	0.598	0.193	1.851	0.3724	0.750	0.239	2.347	0.6207
Pony	1.200	0.652	2.208	0.558	1.260	0.654	2.427	0.4898
Young	1	NA	NA	Ref	1	NA	NA	Ref
Colt/filly	1.189	0.733	1.930	0.4831	1.290	0.764	2.179	0.3412
Adult	0.673	0.516	0.878	0.0035	0.726	0.553	0.953	0.0212
Old	0.416	0.230	0.751	0.0036	0.486	0.265	0.889	0.0193
Female	1	NA	NA	Ref	1	NA	NA	Ref
Gelding	1.051	0.800	1.379	0.7228	0.965	0.708	1.315	0.8224
Male	1.677	1.090	2.581	0.187	1.738	1.119	2.701	0.0139
No activity	--	--	--	--	1	NA	NA	Ref
Driving	--	--	--	--	0.329	0.061	1.781	0.1972
Endurance	--	--	--	--	0.701	0.324	1.515	0.366
English riding	--	--	--	--	1.210	0.797	1.836	0.3717
Show	--	--	--	--	8.760	1.138	67.447	0.0372
Trekking	--	--	--	--	0.892	0.517	1.538	0.68
Western riding	--	--	--	--	1.521	0.900	2.570	0.1169
Other	--	--	--	--	4.436	1.644	11.968	0.0033

**Table 6:** Multivariate analysis to determine risk factors for the severity of ESGD. Comparison between the two models: “base”, with only signalment, and “full”, with signalment and activity. Ref: reference; NA: not available. Likelihood Ratio Test: activity on ESGD; Chi-Square statistic (weight): 45.74; *p*-value: <0.001.

	Base Model, Only Signalment				Full Model, with Activity			
	Odds Ratio	2.5%	97.5%	*p*-Value	Odds Ratio	2.5%	97.5%	*p*-Value
Fullblood	1	NA	NA	Ref	1	NA	NA	Ref
Warmblood	1.007	0.800	1.266	0.9557	1.128	0.838	1.517	0.4281
Baroque	0.681	0.360	1.289	0.2383	0.803	0.409	1.576	0.5229
Coldblood	0.571	0.212	1.539	0.2683	0.707	0.265	1.883	0.4875
Pony	1.286	0.752	2.200	0.3577	1.479	0.831	2.633	0.1833
Young	1	NA	NA	Ref	1	NA	NA	Ref
Colt/filly	1.282	0.860	1.912	0.2226	1.290	0.843	1.974	0.2406
Adult	0.739	0.589	0.927	0.009	0.805	0.639	1.015	0.0671
Old	0.435	0.254	0.745	0.0024	0.482	0.279	0.833	0.009
Female	1	NA	NA	Ref	1	NA	NA	Ref
Gelding	1.055	0.835	1.333	0.6521	0.957	0.737	1.242	0.7404
Male	1.918	1.347	2.729	<0.001	2.032	1.419	2.908	<0.001
No activity	--	--	--	--	1	NA	NA	Ref
Driving	--	--	--	--	0.163	0.029	0.905	0.0381
Endurance	--	--	--	--	0.762	0.378	1.535	0.4467
English riding	--	--	--	--	1.175	0.822	1.681	0.3756
Show	--	--	--	--	4.212	1.670	10.623	0.0023
Trekking	--	--	--	--	1.062	0.660	1.711	0.8036
Western riding	--	--	--	--	1.836	1.195	2.822	0.0056
Other	--	--	--	--	2.660	1.435	4.931	0.0019

**Table 7:** Multivariate analysis to determine risk factors for the presence of EGGD. Comparison between the two models: “base”, with only signalment, and “full”, with signalment and activity. Ref: reference; NA: not available. Likelihood Ratio Test: activity on ESGD; Chi-Square statistic (weight): 37.28; *p*-value: <0.001.

	Base Model, Only Signalment				Full Model, with Activity			
	Odds Ratio	2.5%	97.5%	*p*-Value	Odds Ratio	2.5%	97.5%	*p*-Value
Fullblood	1	NA	NA	Ref	1	NA	NA	Ref
Warmblood	1.874	0.661	1.154	0.3415	0.758	0.521	1.105	0.1494
Baroque	0.858	0.404	1.820	0.6897	0.692	0.311	1.538	0.3663
Coldblood	0.346	0.075	1.605	0.1754	0.374	0.080	1.756	0.2127
Pony	1.234	0.669	2.275	0.5008	1.064	0.544	2.085	0.8555
Young	1	NA	NA	Ref	1	NA	NA	Ref
Colt/filly	1.811	1.167	2.809	0.0081	2.607	1.592	4.270	<0.001
Adult	0.716	0.542	0.947	0.0191	0.761	0.571	1.015	0.0628
Old	0.353	0.161	0.774	0.0094	0.433	0.195	0.963	0.0401
Female	1	NA	NA	Ref	1	NA	NA	Ref
Gelding	1.193	0.896	1.589	0.227	0.950	0.690	1.309	0.7541
Male	1.462	0.974	2.194	0.0668	1.624	1.070	2.467	0.0229
No activity	--	--	--	--	1	NA	NA	Ref
Driving	--	--	--	--	0.602	0.070	5.196	0.6445
Endurance	--	--	--	--	1.159	0.506	2.659	0.727
English riding	--	--	--	--	1.725	1.096	2.715	0.0184
Show	--	--	--	--	0.281	0.086	0.921	0.0361
Trekking	--	--	--	--	1.324	0.733	2.393	0.3525
Western riding	--	--	--	--	1.410	0.844	2.354	0.1898
Other	--	--	--	--	4.327	2.082	8.994	<0.001

**Table 8:** Multivariate analysis to determine risk factors for the presence of ESGD and EGGD at the same time. Comparison between the two models: “base”, with only signalment, and “full”, with signalment and activity. Ref: reference; NA: not available. Likelihood Ratio Test: activity on ESGD; Chi-Square statistic (weight): 28.99; *p*-value: <0.001.

	Base Model, Only Signalment				Full Model, with Activity			
	Odds Ratio	2.5%	97.5%	*p*-Value	Odds Ratio	2.5%	97.5%	*p*-Value
Fullblood	1	NA	NA	Ref	1	NA	NA	Ref
Warmblood	0.922	0.690	1.232	0.5834	0.794	0.536	1.176	0.2495
Baroque	0.781	0.351	1.736	0.5441	0.621	0.268	1.436	0.2652
Coldblood	0.416	0.089	1.937	0.2636	0.427	0.091	2.012	0.2822
Pony	1.089	0.566	2.096	0.7988	0.919	0.450	1.874	0.8154
Young	1	NA	NA	Ref	1	NA	NA	Ref
Colt/filly	1.887	1.212	2.939	0.005	2.585	1.574	4.246	<0.001
Adult	0.661	0.493	0.885	0.0055	0.701	0.519	0.946	0.0201
Old	0.346	0.151	0.792	0.012	0.423	0.183	0.981	0.045
Female	1	NA	NA	Ref	1	NA	NA	Ref
Gelding	1.206	0.894	1.626	0.2194	0.950	0.682	1.321	0.7589
Male	1.607	1.062	2.430	0.246	1.766	1.154	2.702	0.0088
No activity	--	--	--	--	1	NA	NA	Ref
Driving	--	--	--	--	0.816	0.094	7.094	0.8542
Endurance	--	--	--	--	1.298	0.549	3.066	0.5526
English riding	--	--	--	--	1.831	1.137	2.950	0.0128
Show	--	--	--	--	0.350	0.106	1.154	0.0847
Trekking	--	--	--	--	1.534	0.830	2.834	0.1721
Western riding	--	--	--	--	1.485	0.872	2.529	0.1454
Other	--	--	--	--	3.767	1.820	7.795	<0.001

**Table 9:** Results of the full multivariate model for the diagnostic value of clinical signs, signalment, and activity for the presence of ESGD. Ref: reference; NA: not available; AIC: Akaike Information Criterion: 1415.77. Likelihood Ratio Test: Does activity add value to clinical signs? Chi-Square statistic (weight): 43.52; *p*-value: <0.001. Does signalment add value to clinical signs? Chi-Square statistic (weight): 32.83; *p*-value: <0.001. Weight of activity (activity over signalment + signs): Chi-Square statistic (weight): 35.43; *p*-value: <0.001. Weight of signalment (signalment over activity + signs): Chi-Square statistic (weight): 24.74; *p*-value: 0.0033. Added value of clinical signs (signs over signalment + activity): Chi-Square statistic (weight): 40.92; *p*-value: <0.001. The full results can be found in Supplementary Materials Table S3.

	Odds Ratio	2.5%	97.5%	*p*-Value
Fullblood	1	NA	NA	Ref
Warmblood	0.930	0.656	1.318	0.683
Baroque	0.580	0.269	1.252	0.165
Coldblood	0.732	0.225	2.379	0.6041
Pony	1.396	0.719	2.712	0.3242
Young	1	NA	NA	Ref
Colt/filly	1.271	0.748	2.158	0.3751
Adult	0.687	0.520	0.908	0.0082
Old	0.478	0.258	0.887	0.0192
Female	1	NA	NA	Ref
Gelding	0.939	0.685	1.287	0.6947
Male	1.673	1.070	2.614	0.0239
No activity	1	NA	NA	Ref
Driving	0.353	0.065	1.913	0.2271
Endurance	0.673	0.308	1.469	0.32
English riding	1.050	0.687	1.606	0.8216
Show	8.992	1.167	69.276	0.035
Trekking	0.753	0.429	1.322	0.3239
Western riding	1.448	0.853	2.459	0.1706
Other	3.957	1.451	10.760	0.0072
No signs	1	NA	NA	Ref
Clinical signs	4.437	2.648	7.433	<0.001

**Table 10:** Results of the full multivariate model for the diagnostic value of clinical signs, signalment, and activity for the severity of ESGD. Ref: reference; NA: not available; AIC: Akaike Information Criterion: 3407.84. Likelihood Ratio Test: Does activity add value to clinical signs? Chi-Square statistic (weight): 54.62; *p*-value: <0.001. Does signalment add value to clinical signs? Chi-Square statistic (weight): 42.57; *p*-value: <0.001. Weight of activity (activity over signalment + signs): Chi-Square statistic (weight): 47.19; *p*-value: <0.001. Weight of signalment (signalment over activity + signs): Chi-Square statistic (weight): 35.14; *p*-value: 0.0033. Added value of clinical signs (signs over signalment + activity): Chi-Square statistic (weight): 58.81; *p*-value: <0.001. The full results can be found in Supplementary Materials Table S3.

	Odds Ratio	2.5%	97.5%	*p*-Value
Fullblood	1	NA	NA	Ref
Warmblood	1.124	0.833	1.515	0.444
Baroque	0.741	0.376	1.462	0.3877
Coldblood	0.738	0.272	2.003	0.5512
Pony	1.677	0.941	2.989	0.0797
Young	1	NA	NA	Ref
Colt/filly	1.284	0.835	1.974	0.2545
Adult	0.757	0.599	0.957	0.0199
Old	0.476	0.277	0.819	0.0073
Female	1	NA	NA	Ref
Gelding	0.966	0.743	1.256	0.7962
Male	2.053	1.429	2.949	<0.001
No activity	1	NA	NA	Ref
Driving	0.171	0.031	0.957	0.0444
Endurance	0.746	0.371	1.502	0.4121
English riding	1.008	0.702	1.447	0.9676
Show	4.498	1.780	11.366	0.0015
Trekking	0.883	0.545	1.431	0.6146
Western riding	1.778	1.154	2.740	0.0091
Other	2.368	1.269	4.419	0.0067
No signs	1	NA	NA	Ref
Clinical signs	3.848	2.700	5.483	<0.001

**Table 11:** Results of the full multivariate model for the diagnostic value of clinical signs, signalment, and activity for the presence of EGGD. Ref: reference; NA: not available; AIC: Akaike Information Criterion: 1364.58. Likelihood Ratio Test: Does activity add value to clinical signs? Chi-Square statistic (weight): 27.89; *p*-value: <0.001. Does signalment add value to clinical signs? Chi-Square statistic (weight): 38.47; *p*-value: <0.001. Weight of activity (activity over signalment + signs): Chi-Square statistic (weight): 34.39; *p*-value: <0.001. Weight of signalment (signalment over activity + signs): Chi-Square statistic (weight): 44.97; *p*-value: <0.001. Added value of clinical signs (signs over signalment + activity): Chi-Square statistic (weight): 8.56; *p*-value: 0.0034. The full results can be found in Supplementary Materials Table S3.

	Odds Ratio	2.5%	97.5%	*p*-Value
Fullblood	1	NA	NA	Ref
Warmblood	0.751	0.515	1.097	0.1385
Baroque	0.698	0.313	1.553	0.3776
Coldblood	0.359	0.075	1.717	0.1996
Pony	1.134	0.578	2.225	0.7147
Young	1	NA	NA	Ref
Colt/filly	2.598	1.584	4.259	<0.001
Adult	0.742	0.555	0.990	0.0429
Old	0.435	0.195	0.968	0.0413
Female	1	NA	NA	Ref
Gelding	0.936	0.678	1.292	0.6881
Male	1.597	1.050	2.428	0.0286
No activity	1	NA	NA	Ref
Driving	0.624	0.072	5.388	0.668
Endurance	1.147	0.499	2.634	0.7467
English riding	1.603	1.013	2.535	0.0438
Show	0.287	0.088	0.941	0.0394
Trekking	1.214	0.668	2.209	0.5245
Western riding	1.379	0.824	2.305	0.221
Other	4.062	1.948	8.473	<0.001
No signs	1	NA	NA	Ref
Clinical signs	1.800	1.219	2.660	0.0031

**Table 12:** Results of the full multivariate model for the diagnostic value of clinical signs, signalment, and activity for the presence of ESGD and EGGD at the same time. Ref: reference; NA: not available; AIC: Akaike Information Criterion: 3407.84. Likelihood Ratio Test: Does activity add value to clinical signs? Chi-Square statistic (weight): 20.75; *p*-value: <0.001. Does signalment add value to clinical signs? Chi-Square statistic (weight): 42.88; *p*-value: <0.001. Weight of activity (activity over signalment + signs): Chi-Square statistic (weight): 25.80; *p*-value: 0.0011. Weight of signalment (signalment over activity + signs): Chi-Square statistic (weight): 47.94; *p*-value: 0.0033. Added value of clinical signs (signs over signalment + activity): Chi-Square statistic (weight): 10.92; *p*-value: 0.0012. The full results can be found in Supplementary Materials Table S3.

	Odds Ratio	2.5%	97.5%	*p*-Value
Fullblood	1	NA	NA	Ref
Warmblood	0.786	0.530	1.167	0.2326
Baroque	0.626	0.270	1.455	0.2765
Coldblood	0.408	0.0085	1.963	0.2632
Pony	0.990	0.484	2.024	0.9772
Young	1	NA	NA	Ref
Colt/filly	2.574	1.565	4.235	<0.001
Adult	0.687	0.501	0.918	0.0119
Old	0.424	0.183	0.986	0.0462
Female	1	NA	NA	Ref
Gelding	0.935	0.670	1.305	0.6937
Male	1.735	1.133	2.659	0.0113
No activity	1	NA	NA	Ref
Driving	0.852	0.098	7.408	0.8844
Endurance	1.282	0.541	3.034	0.5723
English riding	1.681	1.037	2.724	0.035
Show	0.359	0.109	1.184	0.0925
Trekking	1.388	0.745	2.584	0.3015
Western riding	1.448	0.849	2.469	0.1743
Other	3.502	1.686	7.276	<0.001
No signs	1	NA	NA	Ref
Clinical signs	1.939	1.305	2.882	0.001

**Table 13:** Results of the univariate analysis for the presence of clinical signs. NA: not available.

	Odds Ratio	2.5%	97.5%	*p*-Value
BREED				
Fullblood	1	NA	NA	Reference
Warmblood	1.985	1.341	2.937	<0.001
Baroque	1.807	0.667	4.891	0.2443
Coldblood	1.971	0.423	9.175	0.3872
Pony	0.664	0.199	2.217	0.5053
SEX				
Female	1	NA	NA	Reference
Gelding	1.907	1.290	2.820	0.0012
Male	1.436	0.806	2.559	0.2193
ACTIVITY				
No activity	1	NA	NA	Reference
Driving	0.0	0.00	Inf	0.9882
Endurance	1.485	0.314	7.021	0.618
English riding	4.703	2.442	9.057	<0.001
Show	0.0	0.0	Inf	0.9795
Trekking	4.949	2.144	11.428	<0.001
Western riding	1.946	0.783	4.837	0.1518
Other	4.050	1.404	11.677	0.0096

**Table 14:** Results of the full multivariate model for the relationship between clinical signs and signalment, given the presence of ESGD. Ref: reference; NA: not available; Akaike Information Criterion: 769.29. Likelihood Ratio Test: value of ESGD presence: Chi-Square statistic (weight): 41.43; *p*-value: <0.001. The complete analysis can be found in Supplementary Materials Table S4.

	Odds Ratio	2.5%	97.5%	*p*-Value
ESGD-negative	1	NA	NA	Ref
ESGD-positive	4.379	2.628	7.297	<0.001
Fullblood	1	NA	NA	Ref
Warmblood	1.870	1.226	2.851	0.0036
Baroque	1.700	0.597	4.840	0.3205
Coldblood	2.135	0.430	10.603	0.3538
Pony	0.563	0.164	1.931	0.3611
Young	1	NA	NA	Ref
Colt/filly	1.069	0.506	2.260	0.8608
Adult	1.546	1.030	2.322	0.0354
Old	0.970	0.356	2.643	0.9531
Female	1	NA	NA	Ref
Gelding	1.705	1.131	2.571	0.0108
Male	1.333	0.726	2.447	0.3542

**Table 15:** Results of the full multivariate model for the relationship between clinical signs and signalment, given the severity of ESGD. Ref: reference; NA: not available; Akaike Information Criterion: 758.13. Likelihood Ratio Test: value of ESGD presence: Chi-Square statistic (weight): 58.59; *p*-value: <0.001. The complete analysis can be found in Supplementary Materials Table S4.

	Odds Ratio	2.5%	97.5%	*p*-Value
ESGD grade 0	1	NA	NA	Ref
ESGD grade 1	1.222	0.482	3.095	0.6728
ESGD grade 2	2.446	1.093	5.474	0.0295
ESGD grade 3	5.118	2.225	11.770	<0.001
ESGD grade 4	6.922	3.352	14.296	<0.001
Fullblood	1	NA	NA	Ref
Warmblood	1.834	1.198	2.810	0.0053
Baroque	1.756	0.614	5.017	0.2934
Coldblood	2.425	0.467	12.581	0.2917
Pony	0.528	0.153	1.822	0.3124
Young	1	NA	NA	Ref
Colt/filly	0.995	0.468	2.118	0.9901
Adult	1.559	1.034	2.351	0.0342
Old	0.982	0.357	2.703	0.9721
Female	1	NA	NA	Ref
Gelding	1.748	1.154	2.647	0.0083
Male	1.177	0.635	2.181	0.6042

**Table 16:** Results of the full multivariate model for the relationship between clinical signs and signalment, given the presence of EGGD. Ref: reference; NA: not available; Akaike Information Criterion: 799.56. Likelihood Ratio Test: value of ESGD presence: Chi-Square statistic (weight): 11.15; *p*-value: <0.001. The complete analysis can be found in Supplementary Materials Table S4.

	Odds Ratio	2.5%	97.5%	*p*-Value
EGGD-negative	1	NA	NA	Ref
EGGD-positive	1.932	1.319	2.831	<0.001
Fullblood	1	NA	NA	Ref
Warmblood	1.842	1.219	2.782	0.0037
Baroque	1.568	0.565	4.348	0.3876
Coldblood	2.198	0.464	10.409	0.3211
Pony	0.604	0.179	2.045	0.4182
Young	1	NA	NA	Ref
Colt/filly	0.972	0.956	2.128	0.082
Adult	1.426	0.956	2.128	0.9396
Old	0.874	0.326	2.341	0.7885
Female	1	NA	NA	Ref
Gelding	1.676	1.120	2.509	0.012
Male	1.410	0.769	2.584	0.2665

**Table 17:** Results of the full multivariate model for the relationship between clinical signs and signalment, given the presence of ESGD and EGGD at the same time. Ref: reference; NA: not available; Akaike Information Criterion: 769.29. Likelihood Ratio Test: value of ESGD presence: Chi-Square statistic (weight): 13.29; *p*-value: <0.001. The complete analysis can be found in Supplementary Materials Table S4.

	Odds Ratio	2.5%	97.5%	*p*-Value
EGUS-negative	1	NA	NA	Ref
EGUS-positive	2.088	1.415	3.080	<0.001
Fullblood	1	NA	NA	Ref
Warmblood	1.833	1.213	2.771	0.004
Baroque	1.584	0.569	4.407	0.3783
Coldblood	2.169	0.458	10.282	0.3293
Pony	0.618	0.183	2.092	0.4394
Young	1	NA	NA	Ref
Colt/filly	0.952	0.452	2.006	0.8974
Adult	1.449	0.970	2.165	0.0701
Old	0.877	0.327	2.351	0.7935
Female	1	NA	NA	Ref
Gelding	1.672	1.117	2.505	0.0125
Male	0.388	0.756	2.548	0.2899

## Data Availability

The data presented in this study are available on request from the corresponding author.

## Supplementary Materials

The following supporting information can be downloaded at: https://www.mdpi.com/article/10.3390/ani16182880/s1, Table S1: Results of the univariate models. Ref: reference; NA: not available. Table S2: Results of the multivariate models determining risk factors for the presence of ESGD, EGGD, and both at the same time and for the severity of ESGD. Ref: reference; NA: not available; AIC: Akaike Information Criterion. Table S3: Results of the multivariate models for the diagnostic value of clinical signs, signalment, and activity for gastric ulcers. Ref: reference; NA: not available; AIC: Akaike Information Criterion. Table S4: Results of the multivariate models for the relationship between clinical signs and signalment, given the presence of gastric ulcers. Ref: reference; NA: not available; AIC: Akaike Information Criterion.
