# Supplementary material for: Clinical Signs as an Indication of the Presence of Gastric Ulcers in Horses: True or False?
Source: Animals (Basel). 2026 Sep 13;16(18):2880. doi: 10.3390/ani16182880 (PMC13603533; doi:10.3390/ani16182880)
Supplement: Supplementary file 1 [file animals-16-02880-s001.zip › Table S1.pdf]

Table S1: Results of the univariate models Ref: reference, NA: not available

Presence of ESGD

	Odds Ratio	2.5%	97.5%	p value
BREED				
Fullblood	1	NA	NA	Reference
Warmblood	0.809	0.627	1.043	0.1020
Baroque	0.622	0.312	1.238	0.1762
Coldblood	0.611	0.202	1.844	0.3817
Pony	0.989	0.543	1.800	0.9702
AGE GROUPS				
Young	1	NA	NA	Reference
Colt/Filly	1.330	0.830	2.131	0.2360
Adult	0.658	0.506	0.854	0.0017
Old	0.430	0.240	0.768	0.0044
SEX				
Female	1	NA	NA	Reference
Gelding	1.003	0.770	1.307	0.9823
Male	1.671	1.108	2.521	0.0143
ACTIVITY				
No activity	1	NA	NA	Reference
Driving	0.261	0.050	1.369	0.1121
Endurance	0.738	0.353	1.545	0.4209
English riding	1.155	0.837	1.592	0.3805
Show	13.032	1.722	98.615	0.0129
Trekking	0.824	0.492	1.379	0.4615
Western riding	1.781	1.094	2.900	0.0203
Other	4.431	1.677	11.708	0.0027
PRESENCE OF CLINICAL SIGNS				
No	1	NA	NA	Reference
Yes	3.978	2.407	6.574	<0.001
CATEGORIES OF CLINICAL SIGNS				
No clinical signs	1	NA	NA	Reference
Gastrointestinal signs	3.223	1.663	6.246	0.001
Poor performance	3851444.569	0.000	Inf	0.9678
Weight loss	3.763	1.755	8.067	<0.001

Severity of ESGD

	Odds Ratio	2.5%	97.5%	p value
BREED				
Fullblood	1	NA	NA	Reference
Warmblood	0.881	0.709	1.095	0.2535
Baroque	0.731	0.394	1.357	0.3211

Coldblood	0.621	0.239	1.610	0.3268
Pony	1.046	0.618	1.772	0.8665
AGE GROUPS				
Young	1	NA	NA	Reference
Colt/Filly	1.438	0.975	2.120	0.0670
Adult	0.726	0.581	0.909	0.0051
Old	0.467	0.275	0.792	0.0047
SEX				
Female	1	NA	NA	Reference
Gelding	1.023	0.814	1.284	0.8478
Male	1.925	1.367	2.710	<0.001
ACTIVITY				
No activity	1	NA	NA	Reference
Driving	0.147	0.027	0.786	0.025
Endurance	0.728	0.369	1.436	0.3596
English riding	1.200	0.910	1.583	0.1962
Show	5.574	2.254	13.784	<0.001
Trekking	0.951	0.603	1.501	0.8297
Western riding	1.961	1.317	2.920	<0.001
Other	2.577	1.417	4.684	0.0019
PRESENCE OF CLINICAL SIGNS				
No	1	NA	NA	Reference
Yes	3.384	2.403	4.765	<0.001
CATEGORIES OF CLINICAL SIGNS				
No clinical signs	1	NA	NA	Reference
Gastrointestinal signs	2.551	1.617	4.020	<0.001
Poor performance	12.030	3.437	42.104	<0.001
Weight loss	3.657	2.148	6.224	<0.001

Presence of EGGD

	Odds Ratio	2.5%	97.5%	p value
BREED				
Fullblood	1	NA	NA	Reference
Warmblood	0.760	0.584	0.989	0.0409
Baroque	0.854	0.409	1.782	0.6736
Coldblood	0.339	0.074	1.544	0.1619
Pony	0.986	0.542	1.795	0.9635
AGE GROUPS				
Young	1	NA	NA	Reference
Colt/Filly	1.943	1.270	2.972	0.0022
Adult	0.702	0.533	0.924	0.0116
Old	0.355	0.163	0.771	0.0089
SEX				

Female	1	NA	NA	Reference
Gelding	1.112	0.842	1.468	0.4531
Male	1.593	1.086	2.337	0.0173
ACTIVITY				
No activity	1	NA	NA	Reference
Driving	0.431	0.054	3.897	0.4769
Endurance	1.257	0.566	2.789	0.5744
English riding	1.403	0.994	1.979	0.0542
Show	0.651	0.211	2.002	0.4534
Trekking	1.178	0.672	2.064	0.5682
Western riding	1.999	1.254	3.188	0.0036
Other	4.424	2.192	8.928	<0.001
PRESENCE OF CLINICAL SIGNS				
No	1	NA	NA	Reference
Yes	1.802	1.245	2.607	0.0018
CATEGORIES OF CLINICAL SIGNS				
No clinical signs	1	NA	NA	Reference
Gastrointestinal signs	1.474	0.876	2.479	0.1439
Poor performance	3.449	1.217	9.774	0.0199
Weight loss	1.903	1.089	3.323	0.0237

Presence of ESGD and EGGD at the same time

	Odds Ratio	2.5%	97.5%	p value
BREED				
Fullblood	1	NA	NA	Reference
Warmblood	0.784	0.597	1.030	0.0809
Baroque	0.781	0.358	1.705	0.5348
Coldblood	0.410	0.090	1.872	0.25
Pony	0.831	0.438	1.577	0.5716
AGE GROUPS				
Young	1	NA	NA	Reference
Colt/Filly	2.044	1.333	3.135	0.0011
Adult	0.643	0.482	0.859	0.0027
Old	0.358	0.158	0.812	0.014
SEX				
Female	1	NA	NA	Reference
Gelding	1.131	0.847	1.511	0.4044
Male	1.791	1.213	2.645	0.0034
ACTIVITY				
No activity	1	NA	NA	Reference
Driving	0.595	0.070	5.047	0.6343
Endurance	1.398	0.612	3.191	0.4269
English riding	1.497	1.040	2.155	0.0297

Show	0.840	0.272	2.598	0.7626
Trekking	1.339	0.748	2.398	0.3256
Western riding	2.143	1.321	3.477	0.002
Other	3.759	1.878	7.528	<0.001
PRESENCE OF CLINICAL SIGNS				
No	1	NA	NA	Reference
Yes	1.942	1.335	2.824	<0.001
CATEGORIES OF CLINICAL SIGNS				
No clinical signs	1	NA	NA	Reference
Gastrointestinal signs	1.469	0.861	2.508	0.1587
Poor performance	4.207	1.483	11.933	0.0069
Weight loss	2.150	1.227	3.769	0.0075

Presence of clinical signs

	Odds Ratio	2.5%	97.5%	p value
BREED				
Fullblood	1	NA	NA	Reference
Warmblood	1.985	1.341	2.937	<0.001
Baroque	1.807	0.667	4.891	0.2443
Coldblood	1.971	0.423	9.175	0.3872
Pony	0.664	0.199	2.217	0.5053
AGE GROUPS				
Young	1	NA	NA	Reference
Colt/Filly	0.901	0.442	1.836	0.7737
Adult	1.367	0.927	2.016	0.1151
Old	0.930	0.354	2.445	0.8833
SEX				
Female	1	NA	NA	Reference
Gelding	1.907	1.290	2.820	0.0012
Male	1.436	0.806	2.559	0.2193
ACTIVITY				
No activity	1	NA	NA	Reference
Driving	0.0	0.00	Inf	0.9882
Endurance	1.485	0.314	7.021	0.618
English riding	4.703	2.442	9.057	<0.001
Show	0.0	0.0	Inf	0.9795
Trekking	4.949	2.144	11.428	<0.001
Western riding	1.946	0.783	4.837	0.1518
Other	4.050	1.404	11.677	0.0096
