# Supplementary material for: Clinical Signs as an Indication of the Presence of Gastric Ulcers in Horses: True or False?
Source: Animals (Basel). 2026 Sep 13;16(18):2880. doi: 10.3390/ani16182880 (PMC13603533; doi:10.3390/ani16182880)
Supplement: Supplementary file 1 [file animals-16-02880-s001.zip › Table S2.pdf]

Table S2: Results of the multivariate models to determine risk factors for the presence of ESGD, EGGD and both at the same time and for the severity of ESGD. Ref: reference, NA: not available, AIC: Akaike Information Criterion

Presence of ESGD

Model with only signalment

	Odds Ratio	2.5%	97.5%	p value
Fullblood	1	NA	NA	Ref
Warmblood	0.914	0.699	1.194	0.5076
Baroque	0.587	0.289	1.189	0.1392
Coldblood	0.598	0.193	1.851	0.3724
Pony	1.200	0.652	2.208	0.558
Young	1	NA	NA	Ref
Colt/Filly	1.189	0.733	1.930	0.4831
Adult	0.673	0.516	0.878	0.0035
Old	0.416	0.230	0.751	0.0036
Female	1	NA	NA	Ref
Gelding	1.051	0.800	1.379	0.7228
Male	1.677	1.090	2.581	0.187
No activity	--	--	--	--
Driving	--	--	--	--
Endurance	--	--	--	--
English riding	--	--	--	--
Show	--	--	--	--
Trekking	--	--	--	--
Western riding	--	--	--	--
Other	--	--	--	--

Model with signalment and activity

	Odds Ratio	2.5%	97.5%	p value
Fullblood	1	NA	NA	Ref
Warmblood	0.954	0.678	1.343	0.7893
Baroque	0.598	0.281	1.274	0.1829
Coldblood	0.750	0.239	2.347	0.6207
Pony	1.260	0.654	2.427	0.4898
Young	1	NA	NA	Ref
Colt/Filly	1.290	0.764	2.179	0.3412
Adult	0.726	0.553	0.953	0.0212
Old	0.486	0.265	0.889	0.0193
Female	1	NA	NA	Ref
Gelding	0.965	0.708	1.315	0.8224
Male	1.738	1.119	2.701	0.0139
No activity	1	NA	NA	Ref
Driving	0.329	0.061	1.781	0.1972

Endurance	0.701	0.324	1.515	0.366
English riding	1.210	0.797	1.836	0.3717
Show	8.760	1.138	67.447	0.0372
Trekking	0.892	0.517	1.538	0.68
Western riding	1.521	0.900	2.570	0.1169
Other	4.436	1.644	11.968	0.0033

LRT: Activity on ESGD

Chi-Square statistic (Weight): 36.16

Likelihood Ratio Test p-value: < 0.001

Severity of ESGD

Model with only signalment (ref: reference)

	Odds Ratio	2.5%	97.5%	p value
Fullblood	1	NA	NA	Ref
Warmblood	1.007	0.800	1.266	0.9557
Baroque	0.681	0.360	1.289	0.2383
Coldblood	0.571	0.212	1.539	0.2683
Pony	1.286	0.752	2.200	0.3577
Young	1	NA	NA	Ref
Colt/Filly	1.282	0.860	1.912	0.2226
Adult	0.739	0.589	0.927	0.009
Old	0.435	0.254	0.745	0.0024
Female	1	NA	NA	Ref
Gelding	1.055	0.835	1.333	0.6521
Male	1.918	1.347	2.729	<0.001

Model with signalment and activity (ref: reference)

	Odds Ratio	2.5%	97.5%	p value
Fullblood	1	NA	NA	Ref
Warmblood	1.128	0.838	1.517	0.4281
Baroque	0.803	0.409	1.576	0.5229
Coldblood	0.707	0.265	1.883	0.4875
Pony	1.479	0.831	2.633	0.1833
Young	1	NA	NA	Ref
Colt/Filly	1.290	0.843	1.974	0.2406
Adult	0.805	0.639	1.015	0.0671
Old	0.482	0.279	0.833	0.009
Female	1	NA	NA	Ref
Gelding	0.957	0.737	1.242	0.7404
Male	2.032	1.419	2.908	<0.001
No activity	1	NA	NA	Ref
Driving	0.163	0.029	0.905	0.0381
Endurance	0.762	0.378	1.535	0.4467

English riding	1.175	0.822	1.681	0.3756
Show	4.212	1.670	10.623	0.0023
Trekking	1.062	0.660	1.711	0.8036
Western riding	1.836	1.195	2.822	0.0056
Other	2.660	1.435	4.931	0.0019

Comparison between the two models, base with only signalment and full, with signalment and activity (ref: reference)

	Base model, only signalment				Full model, with activity			
	Odds Ratio	2.5%	97.5%	p value	Odds Ratio	2.5%	97.5%	p value
Fullblood	1	NA	NA	Ref	1	NA	NA	Ref
Warmblood	1.007	0.800	1.266	0.9557	1.128	0.838	1.517	0.4281
Baroque	0.681	0.360	1.289	0.2383	0.803	0.409	1.576	0.5229
Coldblood	0.571	0.212	1.539	0.2683	0.707	0.265	1.883	0.4875
Pony	1.286	0.752	2.200	0.3577	1.479	0.831	2.633	0.1833
Young	1	NA	NA	Ref	1	NA	NA	Ref
Colt/Filly	1.282	0.860	1.912	0.2226	1.290	0.843	1.974	0.2406
Adult	0.739	0.589	0.927	0.009	0.805	0.639	1.015	0.0671
Old	0.435	0.254	0.745	0.0024	0.482	0.279	0.833	0.009
Female	1	NA	NA	Ref	1	NA	NA	Ref
Gelding	1.055	0.835	1.333	0.6521	0.957	0.737	1.242	0.7404
Male	1.918	1.347	2.729	<0.001	2.032	1.419	2.908	<0.001
No activity	--	--	--	--	1	NA	NA	Ref
Driving	--	--	--	--	0.163	0.029	0.905	0.0381
Endurance	--	--	--	--	0.762	0.378	1.535	0.4467
English riding	--	--	--	--	1.175	0.822	1.681	0.3756
Show	--	--	--	--	4.212	1.670	10.623	0.0023
Trekking	--	--	--	--	1.062	0.660	1.711	0.8036
Western riding	--	--	--	--	1.836	1.195	2.822	0.0056
Other	--	--	--	--	2.660	1.435	4.931	0.0019

LRT: Activity on ESGD

Chi-Square statistic (Weight): 45.74

Likelihood Ratio Test p-value: < 0.001

Presence of EGGD

Model with only signalment (ref: reference)

	Odds Ratio	2.5%	97.5%	p value
Fullblood	1	NA	NA	Ref
Warmblood	1.874	0.661	1.154	0.3415
Baroque	0.858	0.404	1.820	0.6897

Coldblood	0.346	0.075	1.605	0.1754
Pony	1.234	0.669	2.275	0.5008
Young	1	NA	NA	Ref
Colt/Filly	1.811	1.167	2.809	0.0081
Adult	0.716	0.542	0.947	0.0191
Old	0.353	0.161	0.774	0.0094
Female	1	NA	NA	Ref
Gelding	1.193	0.896	1.589	0.227
Male	1.462	0.974	2.194	0.0668

Model with signalment and activity (ref: reference)

	Odds Ratio	2.5%	97.5%	p value
Fullblood	1	NA	NA	Ref
Warmblood	0.758	0.521	1.105	0.1494
Baroque	0.692	0.311	1.538	0.3663
Coldblood	0.374	0.080	1.756	0.2127
Pony	1.064	0.544	2.085	0.8555
Young	1	NA	NA	Ref
Colt/Filly	2.607	1.592	4.270	<0.001
Adult	0.761	0.571	1.015	0.0628
Old	0.433	0.195	0.963	0.0401
Female	1	NA	NA	Ref
Gelding	0.950	0.690	1.309	0.7541
Male	1.624	1.070	2.467	0.0229
No activity	1	NA	NA	Ref
Driving	0.602	0.070	5.196	0.6445
Endurance	1.159	0.506	2.659	0.727
English riding	1.725	1.096	2.715	0.0184
Show	0.281	0.086	0.921	0.0361
Trekking	1.324	0.733	2.393	0.3525
Western riding	1.410	0.844	2.354	0.1898
Other	4.327	2.082	8.994	<0.001

Comparison between the two models, base with only signalment and full, with signalment and activity (ref: reference)

	Base model, only signalment				Full model, with activity			
	Odds Ratio	2.5%	97.5%	p value	Odds Ratio	2.5%	97.5%	p value
Fullblood	1	NA	NA	Ref	1	NA	NA	Ref
Warmblood	1.874	0.661	1.154	0.3415	0.758	0.521	1.105	0.1494
Baroque	0.858	0.404	1.820	0.6897	0.692	0.311	1.538	0.3663
Coldblood	0.346	0.075	1.605	0.1754	0.374	0.080	1.756	0.2127
Pony	1.234	0.669	2.275	0.5008	1.064	0.544	2.085	0.8555
Young	1	NA	NA	Ref	1	NA	NA	Ref
Colt/Filly	1.811	1.167	2.809	0.0081	2.607	1.592	4.270	<0.001
Adult	0.716	0.542	0.947	0.0191	0.761	0.571	1.015	0.0628

Old	0.353	0.161	0.774	0.0094	0.433	0.195	0.963	0.0401
Female	1	NA	NA	Ref	1	NA	NA	Ref
Gelding	1.193	0.896	1.589	0.227	0.950	0.690	1.309	0.7541
Male	1.462	0.974	2.194	0.0668	1.624	1.070	2.467	0.0229
No activity	--	--	--	--	1	NA	NA	Ref
Driving	--	--	--	--	0.602	0.070	5.196	0.6445
Endurance	--	--	--	--	1.159	0.506	2.659	0.727
English riding	--	--	--	--	1.725	1.096	2.715	0.0184
Show	--	--	--	--	0.281	0.086	0.921	0.0361
Trekking	--	--	--	--	1.324	0.733	2.393	0.3525
Western riding	--	--	--	--	1.410	0.844	2.354	0.1898
Other	--	--	--	--	4.327	2.082	8.994	<0.001

LRT: Activity on ESGD

Chi-Square statistic (Weight): 37.28

Likelihood Ratio Test p-value: < 0.001

Presence of EGUS

Model with only signalment (ref: reference)

	Odds Ratio	2.5%	97.5%	p value
Fullblood	1	NA	NA	Ref
Warmblood	0.922	0.690	1.232	0.5834
Baroque	0.781	0.351	1.736	0.5441
Coldblood	0.416	0.089	1.937	0.2636
Pony	1.089	0.566	2.096	0.7988
Young	1	NA	NA	Ref
Colt/Filly	1.887	1.212	2.939	0.005
Adult	0.661	0.493	0.885	0.0055
Old	0.346	0.151	0.792	0.012
Female	1	NA	NA	Ref
Gelding	1.206	0.894	1.626	0.2194
Male	1.607	1.062	2.430	0.246

Model with signalment and activity (ref: reference)

	Odds Ratio	2.5%	97.5%	p value
Fullblood	1	NA	NA	Ref
Warmblood	0.794	0.536	1.176	0.2495
Baroque	0.621	0.268	1.436	0.2652
Coldblood	0.427	0.091	2.012	0.2822
Pony	0.919	0.450	1.874	0.8154
Young	1	NA	NA	Ref
Colt/Filly	2.585	1.574	4.246	<0.001
Adult	0.701	0.519	0.946	0.0201

Old	0.423	0.183	0.981	0.045
Female	1	NA	NA	Ref
Gelding	0.950	0.682	1.321	0.7589
Male	1.766	1.154	2.702	0.0088
No activity	1	NA	NA	Ref
Driving	0.816	0.094	7.094	0.8542
Endurance	1.298	0.549	3.066	0.5526
English riding	1.831	1.137	2.950	0.0128
Show	0.350	0.106	1.154	0.0847
Trekking	1.534	0.830	2.834	0.1721
Western riding	1.485	0.872	2.529	0.1454
Other	3.767	1.820	7.795	<0.001

Comparison between the two models, base with only signalment and full, with signalment and activity (ref: reference)

	Base model, only signalment				Full model, with activity			
	Odds Ratio	2.5%	97.5%	p value	Odds Ratio	2.5%	97.5%	p value
Fullblood	1	NA	NA	Ref	1	NA	NA	Ref
Warmblood	0.922	0.690	1.232	0.5834	0.794	0.536	1.176	0.2495
Baroque	0.781	0.351	1.736	0.5441	0.621	0.268	1.436	0.2652
Coldblood	0.416	0.089	1.937	0.2636	0.427	0.091	2.012	0.2822
Pony	1.089	0.566	2.096	0.7988	0.919	0.450	1.874	0.8154
Young	1	NA	NA	Ref	1	NA	NA	Ref
Colt/Filly	1.887	1.212	2.939	0.005	2.585	1.574	4.246	<0.001
Adult	0.661	0.493	0.885	0.0055	0.701	0.519	0.946	0.0201
Old	0.346	0.151	0.792	0.012	0.423	0.183	0.981	0.045
Female	1	NA	NA	Ref	1	NA	NA	Ref
Gelding	1.206	0.894	1.626	0.2194	0.950	0.682	1.321	0.7589
Male	1.607	1.062	2.430	0.246	1.766	1.154	2.702	0.0088
No activity	--	--	--	--	1	NA	NA	Ref
Driving	--	--	--	--	0.816	0.094	7.094	0.8542
Endurance	--	--	--	--	1.298	0.549	3.066	0.5526
English riding	--	--	--	--	1.831	1.137	2.950	0.0128
Show	--	--	--	--	0.350	0.106	1.154	0.0847
Trekking	--	--	--	--	1.534	0.830	2.834	0.1721
Western riding	--	--	--	--	1.485	0.872	2.529	0.1454
Other	--	--	--	--	3.767	1.820	7.795	<0.001

LRT: Activity on ESGD

Chi-Square statistic (Weight): 28.99

Likelihood Ratio Test p-value: < 0.001
