# Supplementary material for: Clinical Signs as an Indication of the Presence of Gastric Ulcers in Horses: True or False?
Source: Animals (Basel). 2026 Sep 13;16(18):2880. doi: 10.3390/ani16182880 (PMC13603533; doi:10.3390/ani16182880)
Supplement: Supplementary file 1 [file animals-16-02880-s001.zip › Table S3.pdf]

Table S3: Results of the multivariate models for the diagnostic value of clinical signs, signalment and activity for gastric ulcers. Ref: reference, NA: not available, AIC: Akaike Information Criterion

Presence of ESGD

Base Model: presence of clinical signs

	Odds Ratio	2.5%	97.5%	p value
Absence	1	NA	NA	Ref
Presence	3.978	2.407	6.574	<0.001

Model 1: Activity and signalment (AIC: 1454.69)

	Odds Ratio	2.5%	97.5%	p value
Fullblood	1	NA	NA	Ref
Warmblood	0.954	0.678	1.343	0.7893
Baroque	0.598	0.281	1.274	0.1829
Coldblood	0.750	0.239	2.347	0.6207
Pony	1.260	0.654	2.427	0.4898
Young	1	NA	NA	Ref
Colt/Filly	1.290	0.764	2.179	0.3412
Adult	0.726	0.553	0.953	0.0212
Old	0.486	0.265	0.889	0.0193
Female	1	NA	NA	Ref
Gelding	0.965	0.708	1.315	0.8224
Male	1.738	1.119	2.701	0.0139
No activity	1	NA	NA	Ref
Driving	0.329	0.061	1.781	0.1972
Endurance	0.701	0.324	1.515	0.366
English riding	1.210	0.797	1.836	0.3717
Show	8.760	1.138	67.447	0.0372
Trekking	0.892	0.517	1.538	0.68
Western riding	1.521	0.900	2.570	0.1169
Other	4.436	1.644	11.968	0.0033

Model 2: Clinical signs + Activity (AIC: 1422.51)

	Odds Ratio	2.5%	97.5%	p value
No activity	1	NA	NA	Ref
Driving	0.273	0.052	1.437	0.1255
Endurance	0.718	0.340	1.514	0.3838
English riding	0.994	0.717	1.379	0.9726
Show	13.669	1.806	103.458	0.0113
Trekking	0.690	0.406	1.173	0.1706

Western riding	1.725	1.056	2.819	0.0295
Other	4.046	1.521	10.760	0.0051
No signs	1	NA	NA	Ref
Clinical signs	4.220	2.533	7.029	<0.001

Model 3: signalment + clinical signs (AIC: 1435.2)

	Odds Ratio	2.5%	97.5%	p value
Fullblood	1	NA	NA	Ref
Warmblood	0.847	0.645	1.113	0.2338
Baroque	0.554	0.269	1.141	0.109
Coldblood	0.546	0.169	1.761	0.311
Pony	1.258	0.680	2.327	0.4654
Young	1	NA	NA	Ref
Colt/Filly	1.176	0.721	1.918	0.5165
Adult	0.638	0.486	0.836	0.0011
Old	0.419	0.229	0.765	0.0046
Female	1	NA	NA	Ref
Gelding	0.974	0.737	1.286	0.8505
Male	1.605	1.038	2.484	0.0335
No signs	1	NA	NA	Ref
Clinical signs	4.401	2.639	7.339	<0.001

Model 4: full model: signalment, activity and clinical signs (AIC: 1415.77)

	Odds Ratio	2.5%	97.5%	p value
Fullblood	1	NA	NA	Ref
Warmblood	0.930	0.656	1.318	0.683
Baroque	0.580	0.269	1.252	0.165
Coldblood	0.732	0.225	2.379	0.6041
Pony	1.396	0.719	2.712	0.3242
Young	1	NA	NA	Ref
Colt/Filly	1.271	0.748	2.158	0.3751
Adult	0.687	0.520	0.908	0.0082
Old	0.478	0.258	0.887	0.0192
Female	1	NA	NA	Ref
Gelding	0.939	0.685	1.287	0.6947
Male	1.673	1.070	2.614	0.0239
No activity	1	NA	NA	Ref
Driving	0.353	0.065	1.913	0.2271
Endurance	0.673	0.308	1.469	0.32
English riding	1.050	0.687	1.606	0.8216
Show	8.992	1.167	69.276	0.035
Trekking	0.753	0.429	1.322	0.3239
Western riding	1.448	0.853	2.459	0.1706
Other	3.957	1.451	10.760	0.0072

No signs	1	NA	NA	Ref
Clinical signs	4.437	2.648	7.433	<0.001

>>> LRT: Does Activity add value to Clinical Signs? <<<
Chi-Square statistic (Weight): 43.52
Likelihood Ratio Test p-value: < 0.001

>>> LRT: Does Signalment add value to Clinical Signs? <<<
Chi-Square statistic (Weight): 32.83
Likelihood Ratio Test p-value: < 0.001

>>> LRT: Weight of Activity (Activity over Signalling+Signs) <<<
Chi-Square statistic (Weight): 35.43
Likelihood Ratio Test p-value: < 0.001

>>> LRT: Weight of Signalment (Signalment over Activity+Signs) <<<
Chi-Square statistic (Weight): 24.74
Likelihood Ratio Test p-value: 0.0033

>>> LRT: Added Value of Clinical Signs (Signs over Signalling+Activity) <<<
Chi-Square statistic (Weight): 40.92
Likelihood Ratio Test p-value: < 0.001

Severity of ESGD

Base Model: presence of clinical signs (AIC: 3463.6)

	Odds Ratio	2.5%	97.5%	p value
Absence	1	NA	NA	Ref
Presence	3.384	2.403	4.765	<0.001

Model 1: Signalment and clinical signs (AIC: 3464.65)

	Odds Ratio	2.5%	97.5%	p value
Fullblood	1	NA	NA	Ref
Warmblood	1.128	0.838	1.517	0.4281
Baroque	0.803	0.409	1.576	0.5229
Coldblood	0.707	0.265	1.883	0.4875
Pony	1.479	0.831	2.633	0.1833
Young	1	NA	NA	Ref
Colt/Filly	1.290	0.843	1.974	0.2406
Adult	0.805	0.639	1.015	0.0671
Old	0.486	0.279	0.833	0.009
Female	1	NA	NA	Ref
Gelding	0.957	0.737	1.242	0.7404

Male	2.032	1.419	2.908	<0.001
No activity	1	NA	NA	Ref
Driving	0.163	0.029	0.905	0.0381
Endurance	0.762	0.378	1.535	0.4467
English riding	1.175	0.822	1.681	0.3756
Show	4.212	1.670	10.623	0.0023
Trekking	1.062	0.660	1.711	0.8036
Western riding	1.836	1.195	2.822	0.0056
Other	2.660	1.435	4.931	0.0019

Model 2: Clinical signs + Activity (AIC: 3424.97)

	Odds Ratio	2.5%	97.5%	p value
No activity	1	NA	NA	Ref
Driving	0.151	0.028	0.814	0.0278
Endurance	0.725	0.368	1.430	0.3538
English riding	1.046	0.790	1.384	0.7554
Show	6.102	2.462	15.121	<0.001
Trekking	0.793	0.501	1.258	0.3249
Western riding	1.942	1.300	2.899	0.0012
Other	2.354	1.285	4.314	0.0056
No signs	1	NA	NA	Ref
Clinical signs	3.643	2.568	5.168	<0.001

Model 3: signalment + clinical signs (AIC: 3439.03)

	Odds Ratio	2.5%	97.5%	p value
Fullblood	1	NA	NA	Ref
Warmblood	0.947	0.751	1.194	0.6459
Baroque	0.602	0.315	1.151	0.1249
Coldblood	0.559	0.204	1.529	0.257
Pony	1.353	0.789	2.319	0.2717
Young	1	NA	NA	Ref
Colt/Filly	1.292	0.863	1.935	0.2134
Adult	0.702	0.558	0.882	0.0024
Old	0.436	0.256	0.744	0.0023
Female	1	NA	NA	Ref
Gelding	1.016	0.804	1.286	0.8917
Male	1.925	1.348	2.749	<0.001
No signs	1	NA	NA	Ref
Clinical signs	3.708	2.615	5.257	<0.001

Model 4: full model (signalment, activity and clinical signs (AIC: 3407.84)

	Odds Ratio	2.5%	97.5%	p value
Fullblood	1	NA	NA	Ref

Warmblood	1.124	0.833	1.515	0.444
Baroque	0.741	0.376	1.462	0.3877
Coldblood	0.738	0.272	2.003	0.5512
Pony	1.677	0.941	2.989	0.0797
Young	1	NA	NA	Ref
Colt/Filly	1.284	0.835	1.974	0.2545
Adult	0.757	0.599	0.957	0.0199
Old	0.476	0.277	0.819	0.0073
Female	1	NA	NA	Ref
Gelding	0.966	0.743	1.256	0.7962
Male	2.053	1.429	2.949	<0.001
No activity	1	NA	NA	Ref
Driving	0.171	0.031	0.957	0.0444
Endurance	0.746	0.371	1.502	0.4121
English riding	1.008	0.702	1.447	0.9676
Show	4.498	1.780	11.366	0.0015
Trekking	0.883	0.545	1.431	0.6146
Western riding	1.778	1.154	2.740	0.0091
Other	2.368	1.269	4.419	0.0067
No signs	1	NA	NA	Ref
Clinical signs	3.848	2.700	5.483	<0.001

>>> LRT: Does Activity add value to Clinical Signs? <<<
Chi-Square statistic (Weight): 54.62
Likelihood Ratio Test p-value: < 0.001

>>> LRT: Does Signalment add value to Clinical Signs? <<<
Chi-Square statistic (Weight): 42.57
Likelihood Ratio Test p-value: < 0.001

>>> LRT: Weight of Activity (Activity over Signalling+Signs) <<<
Chi-Square statistic (Weight): 47.19
Likelihood Ratio Test p-value: < 0.001

>>> LRT: Weight of Signalment (Signalment over Activity+Signs) <<<
Chi-Square statistic (Weight): 35.14
Likelihood Ratio Test p-value: 0.0033

>>> LRT: Added Value of Clinical Signs (Signs over Signalling+Activity) <<<
Chi-Square statistic (Weight): 58.81
Likelihood Ratio Test p-value: < 0.001

Presence of EGGD

Base Model: presence of clinical signs (AIC: 1403.43)

	Odds Ratio	2.5%	97.5%	p value
Absence	1	NA	NA	Ref
Presence	1.802	1.245	2.607	0.0018

Model 1: Activity and clinical signs (AIC: 1371.14)

	Odds Ratio	2.5%	97.5%	p value
Fullblood	1	NA	NA	Ref
Warmblood	0.758	0.521	1.105	0.1494
Baroque	0.692	0.311	1.538	0.3663
Coldblood	0.374	0.080	1.756	0.2127
Pony	1.064	0.544	2.085	0.8555
Young	1	NA	NA	Ref
Colt/Filly	2.607	1.592	4.270	<0.001
Adult	0.761	0.571	1.015	0.0628
Old	0.433	0.195	0.963	0.0401
Female	1	NA	NA	Ref
Gelding	0.950	0.690	1.309	0.7541
Male	1.624	1.070	2.467	0.0229
No activity	1	NA	NA	Ref
Driving	0.602	0.070	5.196	0.6445
Endurance	1.159	0.506	2.659	0.727
English riding	1.725	1.096	2.715	0.0184
Show	0.281	0.086	0.921	0.0361
Trekking	1.324	0.733	2.393	0.3525
Western riding	1.410	0.844	2.354	0.1898
Other	4.327	2.082	8.994	<0.001

Model 2: Clinical signs + Activity (AIC: 1382.97)

	Odds Ratio	2.5%	97.5%	p value
No activity	1	NA	NA	Ref
Driving	0.473	0.056	4.000	0.4919
Endurance	1.243	0.559	2.764	0.5933
English riding	1.301	0.917	1.846	0.1398
Show	0.668	0.217	2.055	0.4812
Trekking	1.086	0.616	1.914	0.7759
Western riding	1.962	1.229	3.133	0.0048
Other	4.198	2.073	8.500	<0.001
No signs	1	NA	NA	Ref
Clinical signs	1.724	1.178	2.521	0.005

Model 3: signalment + clinical signs (AIC: 1382.97)

	Odds Ratio	2.5%	97.5%	p value
Fullblood	1	NA	NA	Ref

Warmblood	0.836	0.631	1.109	0.2139
Baroque	0.831	0.389	1.774	0.6326
Coldblood	0.319	0.067	1.507	0.1491
Pony	1.269	0.686	2.346	0.4478
Young	1	NA	NA	Ref
Colt/Filly	1.813	0.167	2.817	0.0081
Adult	0.697	0.526	0.924	0.012
Old	0.359	0.164	0.788	0.0107
Female	1	NA	NA	Ref
Gelding	1.147	0.858	1.531	0.3544
Male	1.432	0.952	2.152	0.0847
No signs	1	NA	NA	Ref
Clinical signs	1.946	1.329	2.850	<0.001

Model 4: full model: signalment, activity and clinical signs (AIC: 1364.58)

	Odds Ratio	2.5%	97.5%	p value
Fullblood	1	NA	NA	Ref
Warmblood	0.751	0.515	1.097	0.1385
Baroque	0.698	0.313	1.553	0.3776
Coldblood	0.359	0.075	1.717	0.1996
Pony	1.134	0.578	2.225	0.7147
Young	1	NA	NA	Ref
Colt/Filly	2.598	1.584	4.259	<0.001
Adult	0.742	0.555	0.990	0.0429
Old	0.435	0.195	0.968	0.0413
Female	1	NA	NA	Ref
Gelding	0.936	0.678	1.292	0.6881
Male	1.597	1.050	2.428	0.0286
No activity	1	NA	NA	Ref
Driving	0.624	0.072	5.388	0.668
Endurance	1.147	0.499	2.634	0.7467
English riding	1.603	1.013	2.535	0.0438
Show	0.287	0.088	0.941	0.0394
Trekking	1.214	0.668	2.209	0.5245
Western riding	1.379	0.824	2.305	0.221
Other	4.062	1.948	8.473	<0.001
No signs	1	NA	NA	Ref
Clinical signs	1.800	1.219	2.660	0.0031

>>> LRT: Does Activity add value to Clinical Signs? <<<
Chi-Square statistic (Weight): 27.89
Likelihood Ratio Test p-value: < 0.001

>>> LRT: Does Signalment add value to Clinical Signs? <<<
Chi-Square statistic (Weight): 38.47

Likelihood Ratio Test p-value: < 0.001

>>> LRT: Weight of Activity (Activity over Signalling+Signs) <<<
Chi-Square statistic (Weight): 34.39
Likelihood Ratio Test p-value: < 0.001

>>> LRT: Weight of Signalment (Signalment over Activity+Signs) <<<
Chi-Square statistic (Weight): 44.97
Likelihood Ratio Test p-value: <0.001

>>> LRT: Added Value of Clinical Signs (Signs over Signalling+Activity) <<<
Chi-Square statistic (Weight): 8.56
Likelihood Ratio Test p-value: 0.0034

Concurrent presence of ESGD and EGGD

Base Model: presence of clinical signs (AIC: 1326.55)

	Odds Ratio	2.5%	97.5%	p value
Absence	1	NA	NA	Ref
Presence	1.942	1.335	2.824	<0.001

Model 1: Activity and clinical signs (AIC: 1300.31)

	Odds Ratio	2.5%	97.5%	p value
Fullblood	1	NA	NA	Ref
Warmblood	0.794	0.536	1.176	0.2495
Baroque	0.621	0.268	1.436	0.2652
Coldblood	0.427	0.091	2.012	0.2822
Pony	0.919	0.450	1.874	0.8154
Young	1	NA	NA	Ref
Colt/Filly	2.585	1.574	4.246	<0.001
Adult	0.701	0.519	0.946	0.0201
Old	0.423	0.183	0.981	0.045
Female	1	NA	NA	Ref
Gelding	0.950	0.682	1.321	0.7589
Male	1.766	1.154	2.702	0.0088
No activity	1	NA	NA	Ref
Driving	0.816	0.094	7.094	0.8542
Endurance	1.298	0.549	3.066	0.5526
English riding	1.831	1.137	2.950	0.0128
Show	0.350	0.106	1.154	0.0847
Trekking	1.534	0.830	2.834	0.1721
Western riding	1.485	0.872	2.529	0.1454
Other	3.767	1.820	7.795	<0.001

Model 2: Clinical signs + Activity (AIC: 1321.31)

	Odds Ratio	2.5%	97.5%	p value
No activity	1	NA	NA	Ref
Driving	0.614	0.072	5.206	0.6546
Endurance	1.381	0.603	3.159	0.445
English riding	1.373	0.948	1.987	0.0931
Show	0.867	0.280	2.680	0.8037
Trekking	1.220	0.677	2.198	0.5085
Western riding	2.098	1.291	3.409	0.0028
Other	3.534	1.757	7.107	<0.001
No signs	1	NA	NA	Ref
Clinical signs	1.854	1.262	2.724	0.0017

Model 3: Signalment + clinical signs (AIC: 1301.67)

	Odds Ratio	2.5%	97.5%	p value
Fullblood	1	NA	NA	Ref
Warmblood	0.878	0.654	1.177	0.3833
Baroque	0.751	0.335	1.682	0.4862
Coldblood	0.378	0.079	1.804	0.2224
Pony	1.123	0.582	2.170	0.729
Young	1	NA	NA	Ref
Colt/Filly	1.892	1.213	2.952	0.005
Adult	0.639	0.475	0.859	0.003
Old	0.352	0.153	0.807	0.0137
Female	1	NA	NA	Ref
Gelding	1.154	0.853	1.561	0.3529
Male	1.573	1.038	2.384	0.0327
No signs	1	NA	NA	Ref
Clinical signs	2.104	1.428	3.102	<0.001

Model 4: full model: signalment, activity and clinical signs (

	Odds Ratio	2.5%	97.5%	p value
Fullblood	1	NA	NA	Ref
Warmblood	0.786	0.530	1.167	0.2326
Baroque	0.626	0.270	1.455	0.2765
Coldblood	0.408	0.0085	1.963	0.2632
Pony	0.990	0.484	2.024	0.9772
Young	1	NA	NA	Ref
Colt/Filly	2.574	1.565	4.235	<0.001
Adult	0.687	0.501	0.918	0.0119
Old	0.424	0.183	0.986	0.0462
Female	1	NA	NA	Ref
Gelding	0.935	0.670	1.305	0.6937

Male	1.735	1.133	2.659	0.0113
No activity	1	NA	NA	Ref
Driving	0.852	0.098	7.408	0.8844
Endurance	1.282	0.541	3.034	0.5723
English riding	1.681	1.037	2.724	0.035
Show	0.359	0.109	1.184	0.0925
Trekking	1.388	0.745	2.584	0.3015
Western riding	1.448	0.849	2.469	0.1743
Other	3.502	1.686	7.276	<0.001
No signs	1	NA	NA	Ref
Clinical signs	1.939	1.305	2.882	0.001

>>> LRT: Does Activity add value to Clinical Signs? <<<
Chi-Square statistic (Weight): 20.75
Likelihood Ratio Test p-value: 0.0079

>>> LRT: Does Signalment add value to Clinical Signs? <<<
Chi-Square statistic (Weight): 42.88
Likelihood Ratio Test p-value: < 0.001

>>> LRT: Weight of Activity (Activity over Signalling+Signs) <<<
Chi-Square statistic (Weight): 25.8
Likelihood Ratio Test p-value: 0.0011

>>> LRT: Weight of Signalment (Signalment over Activity+Signs) <<<
Chi-Square statistic (Weight): 47.94
Likelihood Ratio Test p-value: 0.0033

>>> LRT: Added Value of Clinical Signs (Signs over Signalling+Activity) <<<
Chi-Square statistic (Weight): 10.92
Likelihood Ratio Test p-value: 0.0012
