# Supplementary material for: Clinical Signs as an Indication of the Presence of Gastric Ulcers in Horses: True or False?
Source: Animals (Basel). 2026 Sep 13;16(18):2880. doi: 10.3390/ani16182880 (PMC13603533; doi:10.3390/ani16182880)
Supplement: Supplementary file 1 [file animals-16-02880-s001.zip › Table S4.pdf]

Table S4: Results of the multivariate models for the relationship between clinical signs and signalment, given the presence of gastric ulcers. Ref: reference, NA: not available, AIC: Akaike Information Criterion

Base model: effect of signalment on presence of clinical signs (AIC: 808.71)

| | Odds Ratio | 2.5% | 97.5% | p value |
|---|---|---|---|---|
| Fullblood | 1 | NA | NA | Ref |
| Warmblood | 1.791 | 1.190 | 2.696 | 0.0052 |
| Baroque | 1.513 | 0.548 | 4.175 | 0.4237 |
| Coldblood | 1.885 | 0.400 | 8.883 | 0.423 |
| Pony | 0.613 | 0.181 | 2.071 | 0.4307 |
| Young | 1 | NA | NA | Ref |
| Colt/Filly | 1.062 | 0.507 | 2.226 | 0.8733 |
| Adult | 1.352 | 0.909 | 2.010 | 0.1365 |
| Old | 0.760 | 0.286 | 2.022 | 0.5828 |
| Female | 1 | NA | NA | Ref |
| Gelding | 1.730 | 1.160 | 2.581 | 0.0072 |
| Male | 1.495 | 0.817 | 2.736 | 0.1924 |

Model 1: effect of signalment on presence of clinical signs, modulated by presence of ESGD (AIC: 769.29)

| | Odds Ratio | 2.5% | 97.5% | p value |
|---|---|---|---|---|
| ESGD negative | 1 | NA | NA | Ref |
| ESGD positive | 4.379 | 2.628 | 7.297 | <0.001 |
| Fullblood | 1 | NA | NA | Ref |
| Warmblood | 1.870 | 1.226 | 2.851 | 0.0036 |
| Baroque | 1.700 | 0.597 | 4.840 | 0.3205 |
| Coldblood | 2.135 | 0.430 | 10.603 | 0.3538 |
| Pony | 0.563 | 0.164 | 1.931 | 0.3611 |
| Young | 1 | NA | NA | Ref |
| Colt/Filly | 1.069 | 0.506 | 2.260 | 0.8608 |
| Adult | 1.546 | 1.030 | 2.322 | 0.0354 |
| Old | 0.970 | 0.356 | 2.643 | 0.9531 |
| Female | 1 | NA | NA | Ref |
| Gelding | 1.705 | 1.131 | 2.571 | 0.0108 |
| Male | 1.333 | 0.726 | 2.447 | 0.3542 |

>>> LRT: Value of ESGD Presence <<<

Chi-Square statistic (Weight): 41.43

Likelihood Ratio Test p-value: < 0.001

Model 2: effect of signalment on presence of clinical signs, modulated by severity of ESGD (AIC: 758.13)

| | Odds Ratio | 2.5% | 97.5% | p value |
|---|---|---|---|---|
| ESGD grade 0 | 1 | NA | NA | Ref |
| ESGD grade 1 | 1.222 | 0.482 | 3.095 | 0.6728 |
| ESGD grade 2 | 2.446 | 1.093 | 5.474 | 0.0295 |
| ESGD grade 3 | 5.118 | 2.225 | 11.770 | <0.001 |
| ESGD grade 4 | 6.922 | 3.352 | 14.296 | <0.001 |
| Fullblood | 1 | NA | NA | Ref |
| Warmblood | 1.834 | 1.198 | 2.810 | 0.0053 |
| Baroque | 1.756 | 0.614 | 5.017 | 0.2934 |
| Coldblood | 2.425 | 0.467 | 12.581 | 0.2917 |
| Pony | 0.528 | 0.153 | 1.822 | 0.3124 |
| Young | 1 | NA | NA | Ref |
| Colt/Filly | 0.995 | 0.468 | 2.118 | 0.9901 |
| Adult | 1.559 | 1.034 | 2.351 | 0.0342 |
| Old | 0.982 | 0.357 | 2.703 | 0.9721 |
| Female | 1 | NA | NA | Ref |
| Gelding | 1.748 | 1.154 | 2.647 | 0.0083 |
| Male | 1.177 | 0.635 | 2.181 | 0.6042 |

>>> LRT: Value of ESGD Presence <<<

Chi-Square statistic (Weight): 58.59

Likelihood Ratio Test p-value: < 0.001

Model 3: effect of signalment on presence of clinical signs, modulated by presence of EGGD (AIC: 799.56)

| | Odds Ratio | 2.5% | 97.5% | p value |
|---|---|---|---|---|
| EGGD negative | 1 | NA | NA | Ref |
| EGGD positive | 1.932 | 1.319 | 2.831 | <0.001 |
| Fullblood | 1 | NA | NA | Ref |
| Warmblood | 1.842 | 1.219 | 2.782 | 0.0037 |
| Baroque | 1.568 | 0.565 | 4.348 | 0.3876 |
| Coldblood | 2.198 | 0.464 | 10.409 | 0.3211 |
| Pony | 0.604 | 0.179 | 2.045 | 0.4182 |
| Young | 1 | NA | NA | Ref |
| Colt/Filly | 0.972 | 0.956 | 2.128 | 0.082 |
| Adult | 1.426 | 0.956 | 2.128 | 0.9396 |
| Old | 0.874 | 0.326 | 2.341 | 0.7885 |
| Female | 1 | NA | NA | Ref |
| Gelding | 1.676 | 1.120 | 2.509 | 0.012 |
| Male | 1.410 | 0.769 | 2.584 | 0.2665 |

>>> LRT: Value of EGGD Presence <<<

Chi-Square statistic (Weight): 11.15
Likelihood Ratio Test p-value: < 0.001

Model 4: effect of signalment on presence of clinical signs, modulated by presence of both ESGD and EGGD (AIC: 769.29)

| | Odds Ratio | 2.5% | 97.5% | p value |
|---|---|---|---|---|
| EGUS negative | 1 | NA | NA | Ref |
| EGUS positive | 2.088 | 1.415 | 3.080 | <0.001 |
| Fullblood | 1 | NA | NA | Ref |
| Warmblood | 1.833 | 1.213 | 2.771 | 0.004 |
| Baroque | 1.584 | 0.569 | 4.407 | 0.3783 |
| Coldblood | 2.169 | 0.458 | 10.282 | 0.3293 |
| Pony | 0.618 | 0.183 | 2.092 | 0.4394 |
| Young | 1 | NA | NA | Ref |
| Colt/Filly | 0.952 | 0.452 | 2.006 | 0.8974 |
| Adult | 1.449 | 0.970 | 2.165 | 0.0701 |
| Old | 0.877 | 0.327 | 2.351 | 0.7935 |
| Female | 1 | NA | NA | Ref |
| Gelding | 1.672 | 1.117 | 2.505 | 0.0125 |
| Male | 0.388 | 0.756 | 2.548 | 0.2899 |

>>> LRT: Value of EGUS status <<<
Chi-Square statistic (Weight): 13.29
Likelihood Ratio Test p-value: < 0.001
